# Genomic, Proteomic and Physiological Characterization of a T5-like Bacteriophage for Control of Shiga Toxin-Producing *Escherichia coli* O157:H7

**DOI:** 10.1371/journal.pone.0034585

**Published:** 2012-04-13

**Authors:** Yan D. Niu, Kim Stanford, Andrew M. Kropinski, Hans-Wolfgang Ackermann, Roger P. Johnson, Yi-Min She, Rafiq Ahmed, Andre Villegas, Tim A. McAllister

**Affiliations:** 1 Lethbridge Research Centre, Agriculture and Agri-Food Canada, Lethbridge, Alberta, Canada; 2 Alberta Agriculture and Rural Development, Agriculture Centre, Lethbridge, Alberta, Canada; 3 Public Health Agency of Canada, Laboratory for Foodborne Zoonoses, Guelph, Ontario, Canada; 4 Department of Cellular and Molecular Biology, University of Guelph, Guelph, Ontario, Canada; 5 Département de microbiologie, Faculté de médecine, Université Laval, Québec, Quebec, Canada; 6 Center for Vaccine Evaluation, Biologics and Genetic Therapies Directorate, Health Canada, Ottawa, Ontario, Canada; 7 Public Health Agency of Canada, National Microbiology Laboratory, Winnipeg, Manitoba, Canada; University of Massachusetts Medical School, United States of America

## Abstract

Despite multiple control measures, *Escherichia coli* O157:H7 (STEC O157:H7) continues to be responsible for many food borne outbreaks in North America and elsewhere. Bacteriophage therapy may prove useful for controlling this pathogen in the host, their environment and food. Bacteriophage vB_EcoS_AKFV33 (AKFV33), a T5-like phage of *Siphoviridae* lysed common phage types of STEC O157:H7 and not non-O157 *E. coli*. Moreover, STEC O157:H7 isolated from the same feedlot pen from which the phage was obtained, were highly susceptible to AKFV33. Adsorption rate constant and burst size were estimated to be 9.31×10^−9^ ml/min and 350 PFU/infected cell, respectively. The genome of AKVF33 was 108,853 bp (38.95% G+C), containing 160 open reading frames (ORFs), 22 tRNA genes and 32 strong promoters recognized by host RNA polymerase. Of 12 ORFs without homologues to T5-like phages, 7 predicted novel proteins while others exhibited low identity (<60%) to proteins in the National Centre for Biotechnology Information database. AKVF33 also lacked the L-shaped tail fiber protein typical of T5, but was predicted to have tail fibers comprised of 2 novel proteins with low identity (37–41%) to tail fibers of *E. coli* phage phiEco32 of *Podoviridae*, a putative side tail fiber protein of a prophage from *E. coli* IAI39 and a conserved domain protein of *E. coli* MS196-1. The receptor-binding tail protein (*pb5*) shared an overall identify of 29–72% to that of other T5-like phages, with no region coding for more than 6 amino acids in common. Proteomic analysis identified 4 structural proteins corresponding to the capsid, major tail, tail fiber and pore-forming tail tip (*pb2*). The genome of AKFV33 lacked regions coding for known virulence factors, integration-related proteins or antibiotic resistance determinants. Phage AKFV33 is a unique, highly lytic STEC O157:H7-specific T5-like phage that may have considerable potential as a pre- and post-harvest biocontrol agent.

## Introduction

Shiga toxin-producing *Escherichia coli* O157:H7 (STEC O157:H7) continues to be one of the major pathogens responsible for foodborne infections in North America [1], [2]. Although it has been nearly 30 years since STEC O157:H7 was first associated with disease in humans, frequent outbreaks as well as sporadic cases continue to occur, with a proportion of cases developing serious acute and chronic sequelae [3].

Healthy cattle are recognized as the primary reservoirs of STEC O157:H7 [4] and up to 30% of North American feedlot cattle shed these bacteria in their feces [4], [5]. Fecal-contaminated meat, fruit, vegetables, and water are the most common infectious sources [3]. Consequently, the majority of mitigation practices developed to date have focused on preventing the adulteration of meat and other foods with STEC O157:H7 during slaughter, processing and retail handling [6]. Another complementary approach to reduce human infections is to mitigate STEC O157:H7 on farm. However, few highly effective on-farm intervention strategies have been developed, although such technologies are being sought [6].

Bacteriophages (phages) are natural infectious agents of bacteria with either lytic or temperate life cycles. Interest in lytic phages as antimicrobial agents is increasing, owing to their target specificity, ability to self replicate and lyse bacteria, and ubiquity within the environment [7], [8]. Phages are being reassessed for their ability to prevent and treat bacterial infections in humans [7], livestock [8], [9] and plants [10] and to decontaminate processed foods and agricultural products [7], [8]. Lytic phages have recently been shown to reduce populations of STEC O157:H7 *in vitro* [11], [12] and within food matrices [13], [14]. However, the complexity and abundant microflora of the ruminant gastro-intestinal tract pose a formidable challenge to the development of phage-based approaches to controlling STEC O157:H7 in ruminants [11], [15]. Phages that specifically infect and kill a broad range of STEC O157:H7 at low multiplicities of infection (MOI) in the intestinal tract or other environments are required to mitigate this human pathogen.

Previously, we found that the presence of natural STEC O157:H7 phages in cattle and the environment are associated with a reduced prevalence of STEC O157:H7 [16]. Here we describe the genomic, proteomic and physiological characteristics of a STEC O157:H7-specific T5-like phage that was frequently isolated during our previous study. We hypothesized that this phage would have unique properties that would be conducive to the selection of phage that would be effective for pre- and post-slaughter control of STEC O157:H7.

## Results

### Phage structure, host range and physiological characteristics

**Figure 1:**
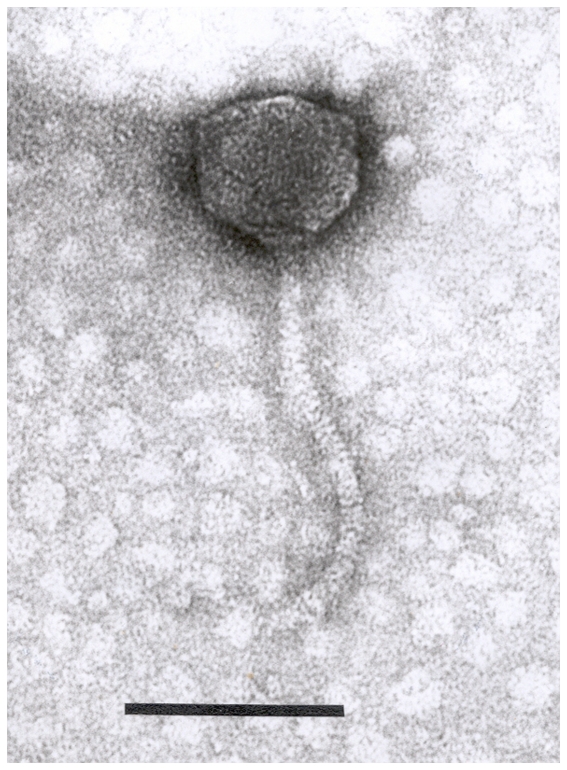
Transmission electron micrograph of AKFV33 negatively stained with uranyl acetate. Scale bar represents 100 nm.

**Figure 2:**
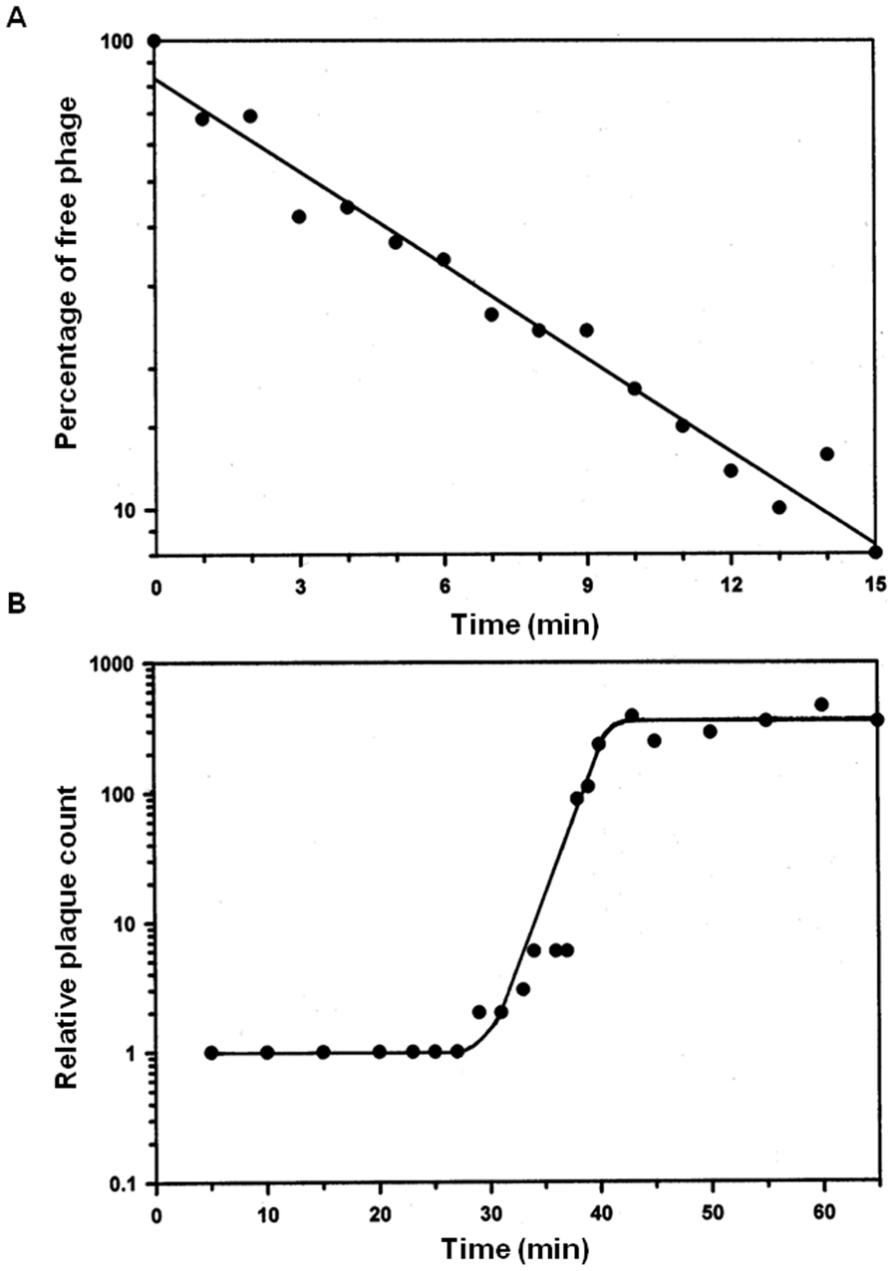
Growth parameters of AKFV33. A, Adsorption curve; B, One step growth curve. Each curve was generated by averaging results from three independent measurements.

Structurally, AKFV33 had an icosahedral head about 78 nm in diameter and a long (185×8.5 nm) rigid, non-contractile, striated tail that ended in a conical structure possessing terminal fibers (23×3 nm, Figure 1). In host range studies, phage AKFV33 was lytic for a wide range of STEC O157:H7 strains and did not lyse any of the non-O157 serotypes of *E. coli* strains examined (Table 1). Of 30 common phage types (PTs) of STEC O157:H7 strains, 27 representing 95% of Canadian STEC O157:H7 isolates (human and livestock) examined in 2007–2009 [2] were highly susceptible [12] to AKFV33 (Avg. MOI = 0.004±0.01). Furthermore, all 27 STEC O157:H7 isolates from the feedlot of origin of AKFV33, five nalidixic acid-resistant strains of human and bovine origin as well as a reference strain EDL933 from a human were lysed by AKFV33 with extremely low MOIs ranging from 10^−5^ to 10^−7^. The adsorption rate constant (*k*) was estimated to be about 9.31×10^−9^ ml/min (Figure 2). The one-step growth curve of AKFV33 propagated on STEC O157:H7 strain R508 in tryptic soy broth (TSB) medium revealed the latent and rise periods were approximately 29 and 14 min (Figure 2), respectively. The average burst size was estimated to be 350 plaque forming units (PFU)/infected cell.

**Table 1:** Host range and lytic capability of phage AKFV33.

Bacteria	Strains	No. of strains	Sensitivity^a^ to AKFV33
STEC O157:H7	Reference strains		
	PTs^b^ 1, 2, 4, 8, 10, 14, 14a, 21, 23, 24, 28, 31, 32, 33, 34, 38, 45, 46, 47, 48, 49, 50, 51, 54, 67, 68, 87	27	MOI = 0.004±0.01
	PTs 63, 70, 74	3	MOI = 3±0.4
	EDL933	1	MOI = 9×10^−6^
	Nalidixic acid-resistant strainsE32511 (PT4), CO281-31N (PT8), R508N (PT14), E318N (PT31), H4420N (PT87)	5	MOI = (6±2)×10^−6^
	Endemic bovine isolates	27	MOI = (7±3)×10^−6^
Non-O157 *E. coli*	ECOR collection^c^ATCC25922 (*E. coli* O6:K−)	73	No lysis observed

a Sensitivity is based on mean multiplicity of infection (MOI: the lowest ratio of phage to bacteria resulting in complete lysis of overnight bacterial culture within 5 h of incubation).

b PTs represents phage types of STEC O157:H7.

c ECOR collection represents standard reference strains of *Escherichia coli* [53].

In acid tolerance studies, titers of phage AKFV33 dropped by 1.99 log_10_ PFU/ml (1.0% survival) after 15 min at pH 3.0 and were undetectable after 2 h, whereas after 16 h at pH 4.5 and 9.3, titres were reduced by 1.09 log_10_ (8.2% survival) and 0.12 log_10_ (76.3% survival) PFU/ml, respectively.

### Genome description

**Figure 3:**
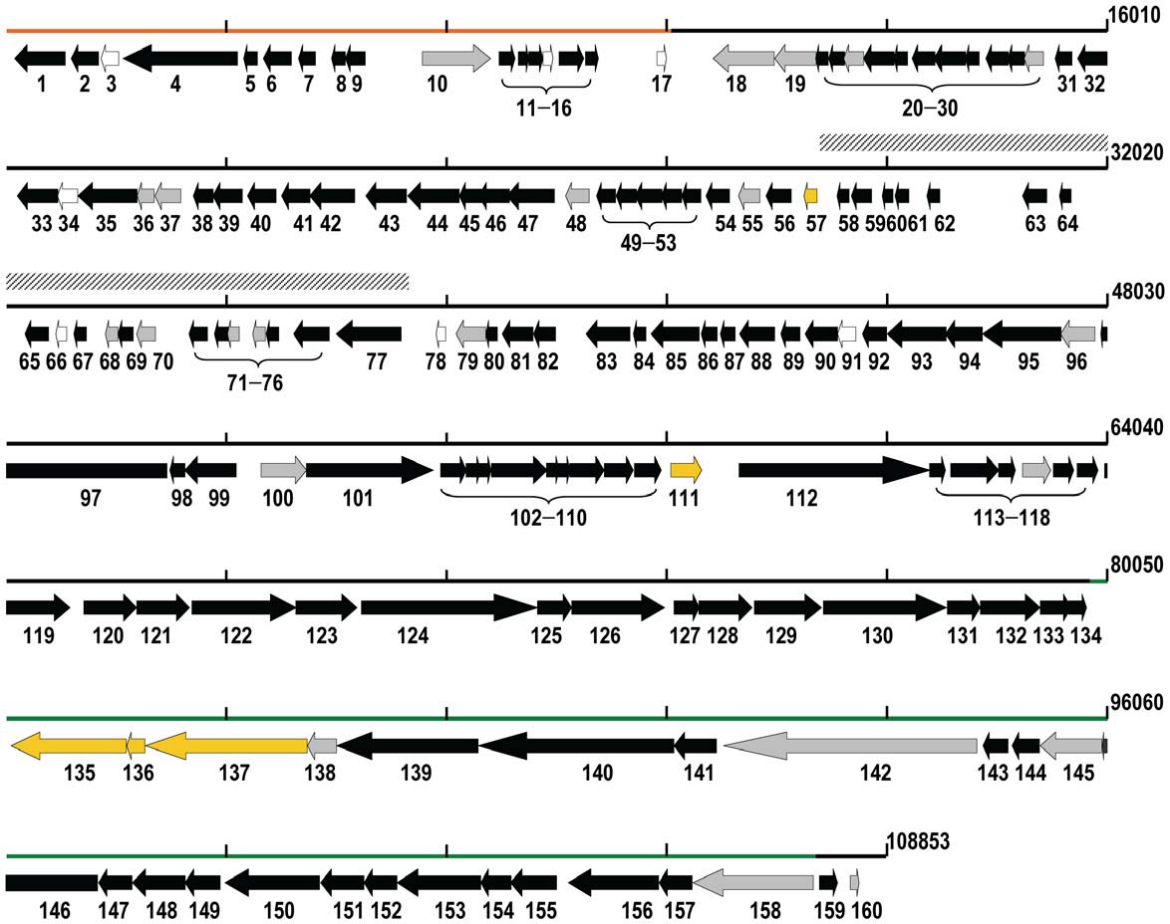
Genomic structure of AKFV33 excluding the terminally redundant DNA sequence. Pre-early, early and late genes regions are indicated by orange, black and green lines, respectively. The predicted ORFs with their directions of transcription are indicated by colored arrows: black, T5-like homologues with greater than 80% identity; gray, T5-like homologues with less than 80% identity; gold, non-T5-like homologues; clear, no homology. The tRNA coding region is indicated by the box with hatched lines.

The AKFV33 genome was ∼111 kb and consisted of linear double-stranded DNA which was sequenced with ∼300 fold coverage (Table 2). The three contigs which made up the total sequence were collapsed using PCR giving a final sequence of 108,853 bp (38.95% G+C). A total of 160 open reading frames (ORFs) and 22 tRNAs (Figure 3 and Figure S1; Table S1), which encoded for 18 amino acids were identified. Furthermore, 19 rho-dependent terminators and 32 promoters recognized by host RNA polymerase were identified. A consensus sequence of the promoters was TTGCTw (−35 box; where “w” = A or T) and TATAATA (−10 box) with a space of 17 to 18 bp between them (Figure S1). Regions of AT-richness were found upstream of −43 region (AAAAATT) and +1 region (TAAATT). Overall, codon usages of phage AKFV33 and STEC O157:H7 EDL933 were correlated (Pearson correlation coefficients *r* = 0.51, *P*<10^−4^, Figure S2).

**Table 2:** General features of phage AKFV33 genome.

Feature	Value
Size (bp)........................................................................................	108,853
Total number of sequences...........................................................	3
G+C content (%)...........................................................................	38.95
A, 29.86; T, 31.20; G, 18.99; C, 19.95	
Total ORFs…………………………………………………….	160
Average ORF size (bp)………………………………………….	586
% of genome coding for proteins……………………………….	86.11
No. of gene products similar to known proteins, total………….	153
No. of gene products similar to known T5, total……………….	148
No. of conserved hypothetical protein with unknown function…	102
No. of hypothetical protein……………………………………...	7
No. of σ^70^ promoters……………………………………………	32
No. of rho-independent terminators…………………………….	19
No. of tRNAs……………………………………………………	22

**Figure 4:**
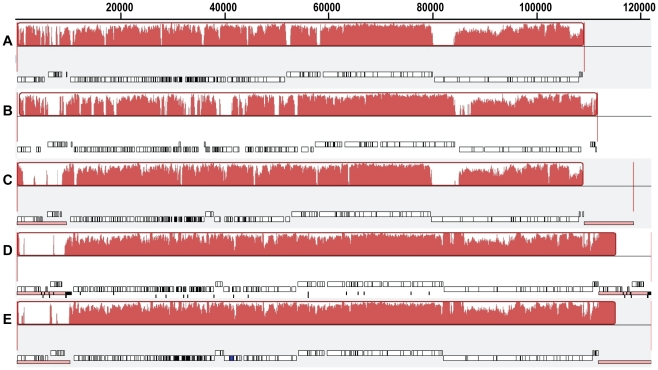
Whole genome comparisons of AKFV33 and other T5-like phages using a progressive MAUVE alignment. The degree of sequence similarity is indicated by the intensity of the red region. The contiguous black boxes under the red region represent the position of the genes. A, AKFV33; B, EPS7; C, SPC35; D, T5 (ATCC11303-B5, GenBank accession#: AY587007); E: T5 (ATCC11303-B5, NC_005859).

In general, AKFV33 had a genome structure in common with T5-like phages including pre-early, early and late gene regions (Figure 3, Table S1). Position-specific iterated (PSI)-BLAST searches indicated that the protein products of 148 ORFs were similar to those of T5. Of the 12 ORFs without homology to T5 genes, 7 predicted completely novel proteins. None of the predicted proteins exhibited homology towards virulence factors, integration-related proteins or antibiotic resistance determinants. Comparative genomic analysis revealed that AKFV33 was collinear with 76–86% nucleotides similar to other members of the T5 group (Figure 4). Computational analysis of CoreGenes showed that AKFV33 shared 126 (75%), 133 (78.2%), 118 (82.5%), 136 (83.9%) and 132 (91%) homologues with phages T5 (GenBank accession #: AY587007), EPS7 (NC_010583), H8 (AC171169), T5 (NC_005859) and SPC35 (HQ406778), respectively.

**Pre-early genes:** The protein products of ORFs 1–16 encoded by pre-early genes in AKFV33 were conserved (Avg. 89% identity) with those of other T5-like phages. Only ORFs 3 and 14 were completely novel. ORFs 1, 4 and 6 encoded deoxynucleoside-5′-monophosphatase (92% identity), phage DNA transfer protein A1 (92% identity) and DNA-binding protein A2 (99% identity), respectively.

**Early genes:** One hundred nineteen ORFs produced proteins which theoretically function early in the phage infection cycle. The majority of these products (111 of 119) displayed an average identity of 89% with T5-like proteins, although 20 exhibited lower identity (38–79%). A high degree of similarity was found for those genes coding for products involved in DNA replication, repair, recombination, transcription, metabolism, cell lysis and signal transduction.

Eight ORFs were predicted to code for essential enzymes associated with nucleotide metabolism: thioredoxin (*nrdC*, ORF38), deoxynucleoside-5′-monosphate kinase (*dnk*, ORF44), thymidylate synthase (*td*, ORF93), dihydrofolate reductase (*frd*, ORF94), small subunit of aerobic ribonucleoside diphosphate reductase (*nrdB*, ORF95) and large subunit of aerobic ribonucleoside diphosphate reductase (*nrdA*, ORF97), anaerobic ribonucleoside triphosphate reductase (*nrdD*, ORF101) and deoxyuridine 5′-triphosphate nucleotidohydrolase (*dut*, ORF133). These eight ORFs exhibited an identity that was 95% of other T5-like phages.

The replication cluster of AKFV33 was also highly-conserved (97% identity) compared to other T5-like phages, including components of the replisome; RNaseH (ORF90), DNA ligase (ORF119 and ORF120), DNA primase (ORF123), helicase (ORF122), DNA polymerase (ORF124), flap endonuclease (ORF132) and a replication origin binding protein (ORF112). Additionally, regions coding for two recombination enzymes D12 (ORF129) and D13 (ORF130) were both highly similar (92% identity) to those of other T5-like phages. However, genes (ORFs 96 and 111) coding for homing endonucleases (HEs) diverged from those reported for T5-like phages. The former, located between *nrdA* and *nrdB*, was 42% identical to H-N-H endonuclease of T5, while the latter had an identity that was only 32% identical to a protein of the *Pseudomonas* podovirus Φ2. ORFs 100 and 155 were predicted to code for a DNA-binding domain whereas ORF121 was predicted to code for a helix-turn-helix XRE domain that corresponded to a T5 phage DNA binding protein, D5 (95% identity).

Three protein products in the early-gene region encoded for a lysozyme (ORF41, 99% identity), holin (ORF42, 95% identity) and cell wall hydrolase SleB (ORF81, 83% identity). The AKFV33 holin, was predicted to be comparatively larger (24.7 kDa) and possess a single transmembrane domain as compared to the 2 to 3 domains that are typical of most phage holins [17].

**Figure 5:**
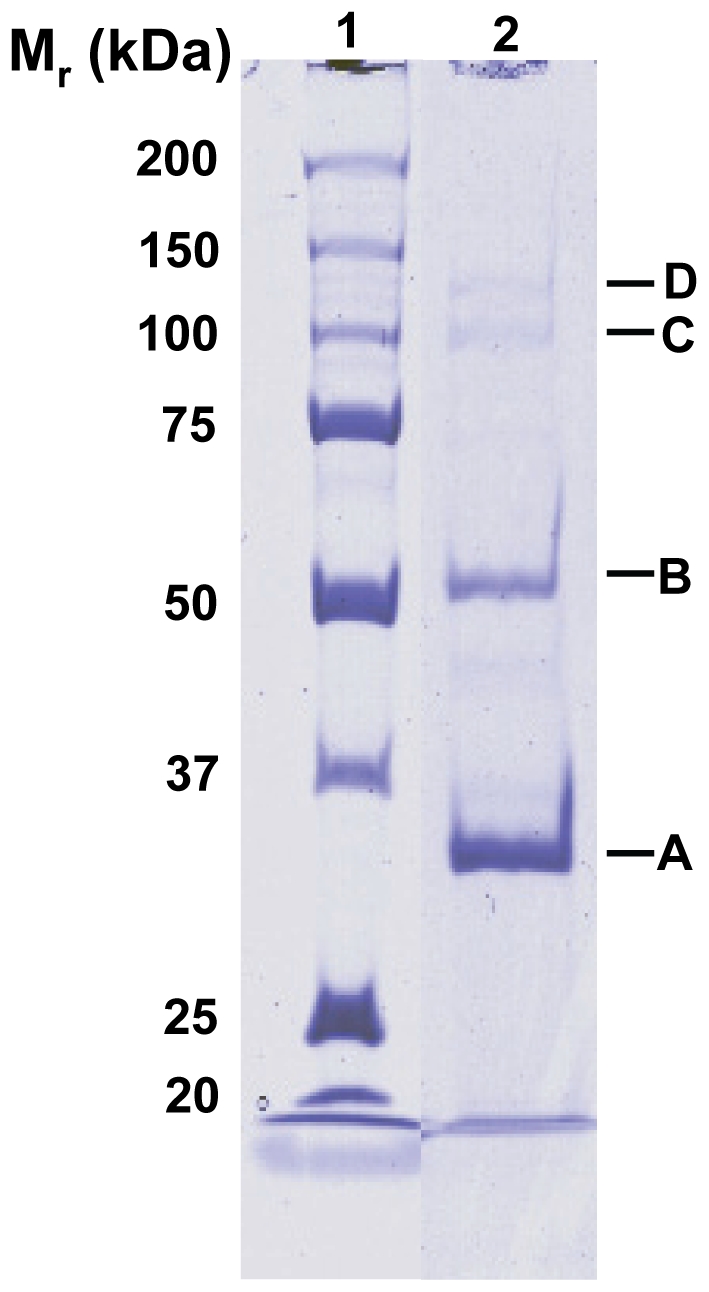
AKFV33 structural proteins (Lane 2) alongside the standard marker (Lane1) separated on 10% SDS-PAGE gel and visualized by Coomassie brilliant blue R250 stain.

**Late genes:** Tail component proteins (ORFs 135, 137–138, 145–146, 152 and *pb2–5*), head related proteins (ORFs 150 and 151), portal protein (ORF153) and terminase (ORF156) were identified in the ORFs 135 to 158. However, the late genes of AKFV33 were the least-conserved with an average identity of 82% as compared to other T5-like phages. Unlike the typical L-shaped tail fiber protein (Ltf) of T5, the tail fiber proteins of AKFV33 were predicted to be encoded for by two gene products associated with ORFs 135 (*ltpB*) and 137 (*ltpA*). Moreover, these two gene products exhibited low predicted identity (37–41%) to proteins in the tail fiber of *E. coli* phage phiEco32 of *Podoviridae* (ORF135), a conserved domain protein of *E. coli* MS196-1 (ORF137), and a putative side tail fiber protein of prophage from *E. coli* IAI39 (ORF137). To further elucidate this regions role in host-specificity, we generated GenBank flat files (*.gbk) extending from the flap endonuclease (D15) to the conserved hypothetical protein (ORF141) among T5-like phages and aligned them using a progressiveMAUVE algorithm (Figure S3). The results clearly showed that the sequence similarity declined markedly for genes encoding tail fibers from T5-like phages. The presence of ORF137 (Table 3 and Table S2; Figure 5, band C) was confirmed as the tail fiber protein by MALDI QqTOF Mass Spectrometry analysis. This gene product was also predicted to belong to the DUF1640 superfamily (cl06708) and with >0.6 probability contained two coiled-coil structures located from residues (249–276) and (314–346). As well, two coiled-coil structures (>0.6 probability) were found in ORF135 (residues 10–38 and 520–557). Similarly, ORF158 (*pb5*) encoded a receptor-binding tail protein of 585 amino acids, which is reputed to be responsible for binding to outer membrane proteins on the host surface during infection. In general, this protein shared only 29–72% identity to that of other T5-like phages (Figure S4). Furthermore, CLUSTALW showed that this receptor binding protein did not share more than 6 amino acids in common with other related T5-like phages (strains D1G, BF23 and ATCC11303-B5, Figure S4).

**Table 3:** Phage AKFV33 structural proteins identified by MALDI QqTOF Mass Spectrometry.

Gel band	ORF	Observed mass (kDa)	Putative function	No. of peptides	Sequence coverage (%)
A	150	32.6	Major head protein	15	44
B	146	51.7	Major tail protein	13	41
C	137	95.8	Tail fiber protein	33	57
D	142	121.1	Pore-forming tail tip protein	23	32

### Proteomics

Mass Spectrometry identified four structural proteins with amino acid sequence coverage up to 57% (Table 3 and S2, Figure 5). The molecular mass of ORF167 (Figure 5, band B) was 51.7 kDa, being close to the predicted size of 50.5 kDa and in agreement with 51 kDa reported by Zweig and Cummings [18].

A major head protein precursor of 32.6 kDa (Figure 5, band A) encoded by ORF150, was substantially smaller than its predicted theoretical size of 50.6 kDa. The mass spectrometry was able to recover 15 peptides located near the C-terminus of the protein precursor, accounting for 44% of the overall amino acid sequence (Table S2). This observation suggests that a truncated form of the mature head protein likely arose as a result of proteolytic cleavage at the N-terminus during head morphogenesis of AKFV33. Others have similarly observed that post-translational processing of the T5 head protein produces a 32 kDa protein [19].

A pore-forming tail tip protein (*pb2*; Figure 5, band D) was observed to have a molecular mass of 121.1 kDa, similar to the 123.9±0.1 kDa of Pb2 previously obtained from T5 phage [20]. A larger theoretical molecular weight (132.3 kDa) and no coverage of peptides from Arg (R^1134^) to C-terminus may indicate that this protein also underwent cleavage during morphogenesis [19]. Similarly, Boulanger *et al.* [20] observed that maturation of Pb2 occurred from the C-terminal end with cleavage after Val^1148^. In addition, ORF142 possessed identical amino acid sequences at both the N-terminus and C-terminus to that of Pb2 in phage T5 (Figure S5), despite these proteins only exhibiting an overall sequence identity of 59%. The secondary protein structure of Pb2 from AKFV33 was predicted to be similar to that of phage T5. Firstly, region I (residues 1–1045) contained 7 regions of coiled-coil structure with >0.6 probability (residues 18–46, 446–473, 577–606, 678–763, 776–804, 846–903, 1004–1045). Following this coiled-coil region, there were 2 regions (residues 1037–1059 and 1061–1083) that were indicative of helical transmembrane spanning segments as illustrated by SOSUI. Lastly, the C-terminal region was predicted to have a zinc metallopeptidase motif located between residues 1142 to 1164.

## Discussion

This study is the first systematic *in-vitro* investigation and evaluation of the eligibility of a T5-like phage for therapeutic application against STEC O157:H7. Morphological, genomic and proteomic analyses indicated that AKFV33 is a T5-like phage belonging to the family *Siphoviridae*, and lacks genes coding for virulence factors and generalized transduction. AKFV33 was active against a broad range of STEC O157:H7, including reference strains of 30 common PTs and 27 bovine field isolates. Yet unlike other STEC O157:H7-infecting phages [14], [21], [22], [23], [24], AKFV33 did not target non-O157 *E. coli*. A relative high specificity for non-target *E. coli* of therapeutic phages is a key factor to determine safe application in the mitigation of STEC O157:H7 (Niu *et al*., unpublished data). Most importantly, AKFV33 exhibited strong lytic activity against virtually all STEC O157:H7 isolates tested.

### General genomic features

The genome of phage AKFV33 displayed a higher degree of identity to genes from T5-like phages encoding for DNA replication, metabolism, recombination and repair as compared to those coding for structural function. This confirms that T5 phages conserve those genes involved in these cellular functions [25]. However, there are discrepancies among HEs between AKFV33 and T5 phage (ATCC11303-B5). Only two HEs (ORFs 96 and 111) were identified in the AKFV33 genome, compared to the nine predicted in phage T5 (ATCC11303-B5, GenBank accession#: NC_005859). Similarly, a T5-like phage, SPC35 with activity against *Salmonella* and *E. coli* exhibited the highest nucleotide similarity (86%) to AKFV33 and was found to possess genes encoding for two HEs [26], while another T5-like phage EPS7, only possessed a single HE [27]. To date, only three HEs belonging to H-N-H subfamily have been revealed to be located in the tRNA gene region of T5 phage (ATCC11303-B5) [28]. All three of these were not identified in the genome of AKFV33. Moreover, the two HEs of AKFV33 were neither closely related to putative H-N-H-endonuclease P-TflVIII of T5 nor that of the podovirus of *Pseudomonas* phage Φ2. Introns or inteins associated HEs and non-introns/inteins-encoding freestanding HEs are mobile genetic elements that can promote the homing of their own genetic elements into allelic intronless and inteinless sites [29]. Freestanding HEs are more abundant than introns/interns associated HEs in T-even phages [30]. These proteins promote gene duplication and protection of DNA from cleavage, but they are not essential and enter a dynamic cycle of invasion, fixation, inactivation, elimination, and eventually reinvasion [29]. Most HEs have elements that improve gene mobility, maximize insertion site availability, and thereby minimize their probability of being lost from the genome [29]. ORF96 is a counterpart of putative H-N-H-endonuclease P-TflVIII of phage T5 which divides the gene of ribonucleotide reductase into *nrdA* and *nrdB*. Moreover, the *nrdA*-*nrdB* region appears to be hotspot for HEs insertion in T-even phages, since *mobE*, a freestanding H-N-H endonuclease, has been discovered to lie between the genes of *nrdA* and *nrd*B in several T-even phages [31]. The similar *nrdA*-*nrdB* splicing H-N-H endonuclease in AKFV33 merits further investigation of its nature and function as it may be similar to *mobE*.

Compared to other T5-like phages, the genome of AKFV33 mainly differed in those structural proteins responsible for host recognition and adhesion. This variation could be responsible for the high specificity of AKFV33 for STEC O157:H7 strains as compared to other T5-like phages [21], [25], [26], [27], [32] and non-T5 like STEC O157:H7-infecting phages [14], [22], [23], [24]. Host range is controlled by the interaction of the phage with receptors on the host cell surface [33]. Host infection and adsorption of phage T5 is initiated by reversible binding of the long tail fibers to the O-antigen of the lipopolysaccharide [34], followed by irreversible binding to outer membrane proteins of FhuA [35] or FepA [32] or BtuB [26] via Pb5. Comparative analysis of the genes and gene products of FhuA, FepA and BtuB using BLASTN and CLUSTALW, respectively, shows that receptor proteins are highly conserved (98–100% identity, BLASTN) among STEC O157:H7 and generic *E. coli*. However, the Pb5 of AKFV33 was not typical of other receptor binding proteins described in T5 phage [25], [26], [27]. Presumably, the tail fiber protein of this phage was highly specific for the O antigen of STEC O157:H7 isolates, but lacked recognition of O antigen exhibited by the cells of non-O157 *E. coli* strains. Further investigation of host receptors would be required to determine whether co-recognition between tail fiber and Pb5 of this phage occurs when infecting hosts, and if cell surface structures other than FhuA, FepA and BtuB influence recognition and binding of Pb5.

### Efficacy in biocontrol

To be efficacious, a phage must exhibit high affinity and lytic activity against a variety of strains of the targeted host bacterium. In most cases, failure of phage to attack the host is attributable to a lack of adsorption [33]. Phages with broad spectrum activity against a wide range of STEC O157:H7 isolates could be advantageous in that fewer phages would be required to control this pathogen over a wider range of production environments. Scale-up and production of the phage for commercial applications would also be simplified.

After adsorption, phage DNA must be introduced into the host through the bacterial cell wall [33]. Boulanger *et al.* [20] demonstrated that Pb2 of T5 phage undergoes a conformational change upon binding to the host and possesses fusogenic and muralytic activities that aide in the introduction of DNA into the host. It is probable that the Pb2 of AKFV33 plays a similar role during host infection, as the coiled-coil region of Pb2 could undergo a conformational shift resulting in the formation of a head-tail connector, enabling the C-terminal region to access the host envelope to form a pore in the inner and outer membrane through which phage DNA could be introduced.

Phages with strong lytic capability eliminate pathogens more rapidly and efficiently [36]. Adsorption rate, latent period and burst size are factors that determine the proliferation rate of a given phage [37]. These biological parameters depend on phage type, host physiology and nutritional conditions [33]. Nevertheless, in the present study all STEC O157:H7 screened were highly susceptible [12] to infection by AKFV33. The conserved nature of pre-early region in AKFV33 suggests that it is capable of rapidly shutting off synthesis of STEC O157:H7 DNA, RNA and proteins, degrading host DNA and inactivating host enzymes such as *EcoR*I, *recBC*, DNA methylase and uracil-DNA glycosylase [38]. T5-like phage introduce DNA into their host in a two-step process, with the first step involving the inactivation of host defenses and the second step the replication of phage DNA [38]. The first step involves the introduction of DNA that encodes gene products such as deoxynucleoside-5′-monophosphatase that ensure deoxynucleotides derived from host DNA are eliminated from the cell prior to the second-step transfer of DNA and the initiation of phage DNA synthesis. Although the mechanisms of two-step DNA transfer in T5-like phages remains unclear, it is imperative that the pre-early mechanisms inactivate host defenses prior to transfer of early and late genes if successful phage replication is to occur [39].

Unlike phages T7 and T4, in which 90% [40], [41] and 20% [42], [43], respectively, of the deoxynucleotides of the phage DNA are derived from the host DNA, T5 phages do not incorporate nucleotides derived from host DNA into their genome, rather they produce nucleotides almost exclusively by *de novo* synthesis [39]. As a result, T5 is the only T-odd phage identified to date that synthesizes considerably more DNA than is initially present in host at time of infection [39]. Like phage T5, the genome of AKFV33 was found to encode 8 of 11 common enzymes [25] required to generate a pool of nucleotides for DNA synthesis.

T5-like phages contain some of the most efficient promoters characterized and exhibit a high affinity for host RNA polymerase [38]. Phage AKFV33 possessed 32 T5-like promoters plus AT rich regions both around −43 regions around and downstream of +1. Promoters that possess AT-rich regions confer curvature to the DNA strand and increase the stability of the RNA polymerase-promoter complex, thereby increasing transcriptional efficiency [44], [45]. Although phages require most of the host's translation apparatus for protein synthesis [46], the overall similar codon usage between AKFV33 and the host would promote efficient translation of gene products [46]. Combined, these properties may contribute to the high transcriptional and translation efficiency and indirectly the high burst size and the low MOI of AKFV33.

As compared to its host STEC O157:H7, the G+C content of AKFV33 was ∼11% lower, a difference similar to that reported for other T5-like phages [25], [26], [27]. Generally, the G+C content tends to be lower in phages than their host due to the reduced availability and higher energetic cost for the synthesis of G and C as compared to A and T/U [47]. Phage DNA typically encodes for tRNAs that when transcribed supplement the host tRNA pools [46]. Presumably, the 22 tRNAs coded for by AKFV33 would compensate for any deficiency of host tRNAs during translation. In phage T5, tRNA genes are expressed from about 5 min after infection until lysis, and are employed in the translation of both early and late transcripts [39].

### Safety

As phages are biological entities capable of transferring genetic material among bacterial genomes, safety is major concern for their therapeutic application either on farm or in the food chain. All T5-like phages presently identified are exclusively lytic with none exhibiting lysogenic characteristics [25], [26], [27], [32]. Genomic and proteomic analysis of the genome of AKFV33 identified no sequence that encoded for virulence factors, integration-related proteins or genes coding for antibiotic resistance. As Phage T5 do not utilize host nucleotides for DNA synthesis and the host DNA are degraded and excreted out of the host cell via first-step transfer of DNA [39], the chances of AKFV33 acquiring virulent genes from STEC O157:H7 are remote.

Phage AKFV33 was selected from naturally occurring phages obtained from commercial feedlot cattle. Phage isolates endemic to the environment to which therapy will be applied may result in more efficacious therapeutic outcomes than phage isolated from other environments [21], [48]. However, AKFV33 is acid-sensitive a property shared with many other therapeutic phage candidates [49], [50], [51]. Consequently, the acidic environment of the stomach may lower phage infectivity [8], [9] and acid protection through encapsulation may be necessary for therapeutic application of the phage in livestock.

On-farm strategies of controlling STEC O157:H7 in livestock and their environment have been marginally successful. The present study demonstrates that unique T5-like phage exhibiting specific and high lytic activity against STEC O157:H7 exist within the feedlot environment. Natural fluctuations in STEC O157:H7 populations in the farm environment may be mediated through changes in phage activity. Identification of novel genes that confer a high and specific affinity of T5-like phage for STEC O157:H7 could serve as the foundation for the selection and formulation of phage cocktails to mitigate this pathogen on farm, in the environment and in food.

## Materials and Methods

### Bacteriophage, bacteria and media

Phage AKFV33 infecting STEC O157:H7 strain R508 (phage type 14, PT14) was isolated from feces from steers housed in a feedlot in 2007 in Alberta, Canada [16]. It was frequently associated with pens of cattle that exhibited reduced prevalence of STEC O157:H7 [16]. A single discrete plaque was purified three times by the soft agar (0.6%) overlay method [52]. Purified phage were then mixed thoroughly with 0.9 ml of sterile lambda diluent (10 mM Tris-HCl, pH 7.5, 8 mM MgSO_4_) and held at room temperature for 1–2 h to allow phage particles to diffuse from the agar. Diluents containing phage were centrifuged at 10, 000×*g* for 10 min and filtered through 0.22-µm syringe filter (Pall, Newquay, Cornwall, UK) to remove agar and bacterial cells. The filtrate was used for preparation of working stocks as follows: 100 µl of phage filtrate were mixed with 1 ml of a mid-log-phase culture of STEC O157:H7 strain R508 (OD_600_: 0.4 to 0.5) and incubated for 15 min at 37°C to enable attachment. This was followed by addition of 250 ml of tryptic soy broth (TSB) amended with MgSO_4_ at 10 mM, and further incubation at 37°C for 4 to 6 h with shaking (170 rev/min) until complete lysis occurred. The lysates was then centrifuged at 5,250×*g* for 20 min at 4°C and filtered through a 0.2-µm SFCA serum filter (Nalgene, Rochester, NY, USA).

Titers of phages in stock filtrates were determined by the soft agar overlay technique [52]. STEC O157:H7 strain R508 was used as a host for plaque purification and titration of phage stocks. Other standard laboratory strains of STEC O157:H7 (n = 36) and non-O157 *E. coli* (n = 73) [53] used to evaluate host range of AKFV33 are listed in Table 1. Bovine STEC O157:H7 strains (n = 27) endemic to the feedlot pen from which the phage was isolated were grown concurrently with phage AKFV33 as previously described [16]. Unless otherwise indicated strains were grown in TSB and/or tryptic soy agar (TSA).

### Host range and lytic capability

Host range and lytic capability of the phage for STEC O157:H7 was assessed using a microplate phage virulence assay [12]. To estimate multiplicity of infection (MOI), high titre phage stocks (10^9^–10^10^ PFU/ml) were serially diluted and incubated at 37°C for 5 h with 10-fold diluted overnight cultures of STEC O157:H7 in a 96-well microplate. After incubation, wells were examined visually for turbidity and the highest dilution that resulted in complete lysis (no discernable turbidity) of bacteria was recorded. The MOI for each phage-host assay was calculated by dividing the initial number of phages in the highest-dilution wells by the initial number of bacteria added, as determined by plate counts of the serially diluted bacterial cultures.

Host specificity for seventy-three non-O157 *E. coli* strains was determined using spot assays. Briefly, each strain of non-O157 *E. coli* in mid-log-phase (OD_600_: 0.3 to 0.4) was spread onto modified nutrient agar (MNA) containing NaCl (8.5 g/l), CaCl_2_ (8.3 mg/l), FeCl_3_ (1.15 mg/l), MgSO_4_ 7H_2_O (0.5 g/l) and 30% (w/v) glucose (10 ml/l) to obtain confluent growth. After the bacterial lawn was dried, 10 µl of phage stock (∼10^6^ PFU/ml) was spotted on MNA and incubated overnight at 37°C. After incubation, each spot was examined for the presence of clearing zones within the bacterial lawn. Sensitivity of the bacteria to the phage was defined as clearing of >50% of the bacterial spot lawn.

### Transmission electron microscopy

Filtered phage lysates (∼10^9^ PFU/ml) were centrifuged at 25,000×*g* for 1 h using an Beckman J2–21 high-speed centrifuge (Palo Alto, CA, USA) and a JA-18.1 fixed angle rotor, followed by two washings in ammonium acetate buffer (0.1 M, pH 7). Phage particles were deposited on copper grids with carbon-coated Formvar films, stained with 2% uranyl acetate (pH 4.5) and examined in a Philips EM 300 electron microscope.

### Growth parameters

Phage attachment assays were conducted thrice using the standard method of Kropinski [54] with minor modifications. A mid-log-phase culture of STEC O157:H7 R508 (OD_600_: 0.3 to 0.4) was mixed with phage lysates to give a MOI of 0.1 and incubated at 37°C. Aliquots (0.2 ml) were taken at 1 min intervals for 15 min and diluted in 19.8 ml of sterile phosphate buffer solution (PBS, pH 7.2, 4°C). The samples were centrifuged at 10,000×*g* for 10 min at 4°C and the supernatants were tested for unabsorbed phages through titration using the overlay technique [52]. The adsorption rate constant (*k*) was calculated.

One-step growth assays were performed thrice using a standard method [37] with modification. A mid-log-phase culture of STEC O157:H7 R508 (OD_600_: 0.3 to 0.4) was infected by a phage lysates to achieve a MOI of 0.1 and incubated at 37°C for 5 min. The phage-host mixture was centrifuged at 10,000×*g* for 10 min at 4°C to remove unabsorbed phages. The pellet containing infected cells was then diluted 1,000- and 20,000-fold, using TSB with 10 mM MgSO_4_ to a final volume of 10 ml and 20 ml, respectively. The diluted phage-bacteria culture (0.1 ml) was withdrawn immediately to estimate initial phage titre (time zero), and the remainder was incubated at 37°C for one-step growth determination. Samples (0.1 ml) were periodically taken over 60 min, diluted and titrated immediately using the overlay technique [52] and the latent period, the rise period and the burst size was calculated.

### pH tolerance assay

Filtered phage lysate (100 µl; ∼10^9^ PFU/ml) was added to 900 µl of TSB (pH 7.2) and adjusted to a pH of 3.0, 4.5 and 9.3 using either HCl (6 M) or NaOH (6 M) and incubated at 37°C for 16 h. Subsamples (20 µl) were then taken periodically for standard plaque assays [52]. Phage activity was examined in triplicate assays for each pH.

### CsCl density gradient centrifugation

Bacterial nucleic acids were removed from filtered phage lysates (∼10^9^ PFU/ml) using DNase1 (Sigma-Aldrich, Oakville, ON, Canada) and RNaseA (Sigma-Aldrich, Oakville, ON, Canada), and the phage lysates were concentrated in polyethylene glycol (PEG) 8000 and purified through two rounds of CsCl density gradient centrifugation [52].

### Genome size estimation

CsCl-purified phages were subjected to pulsed field gel electrophoresis (PFGE) to estimate genome size [55]. Phage DNA embedded in 1% SeaKem Gold agarose (Lonza, Shawinigan, QC, Canada) was subjected to electrophoresis in 0.5×Tris-borate EDTA buffer at 14°C for 18 h using a Chef DR-III Mapper electrophoresis system (Bio-Rad, Mississauga, ON, Canada), with pulse times of 2.2–54.2 s, 6 V/cm. Molecular weight markers were prepared using *Salmonella braenderup* H9812 digested by *Xba*I (Invitrogen, Burlington, ON, Canada). Banding patterns were viewed with UV illumination, and photographed using the Speedlight Platinum Gel Documentation System (BioRad, Mississauga, ON, Canada). All PFGE results were analyzed using BioNumerics software (Applied Maths, Austin, TX, USA).

### Genome sequencing and annotation

Phage DNA was extracted from the CsCl-purified phage lysates using the SDS-proteinase K protocol of Sambrook and Russell [52]. Purified phage DNA was submitted to the McGill University and Génome Québec Innovation Centre (Montréal, QC, Canada) and sequenced using 454 Technology by GS FLX Titanium series (Roche Diagnostics, Laval, QC, Canada). The sequence of this phage resided in three large contigs which were closed by PCR.

The genome was initially subjected to automated annotation using AutoFACT [56], with open reading frames (ORFs) subsequently confirmed using Kodon version 2.0 (Applied Maths, Austin, TX, USA). Predicted translated proteins were scanned for homologues using BLASTP and PSI-BLAST [57], [58] at http://www.ncbi.nlm.nih.gov. Transfer RNA (tRNA)-encoding genes were screened using Aragorn at http://130.235.46.10/ARAGORN/ [59] and tRNAScan at http://greengene.uml.edu/programs/FindtRNA.html [60]. Rho-independent terminators were identified using MFold [61] and TransTerm [62] at http://nbc11.biologie.uni-kl.de/framed/left/menu/auto/right. Promoters were identified by visual inspection and by a neural network prediction programme at http://www.fruitfly.org/seq_tools/promoter.html. Consensus sequences of the promoters were plotted with WebLogo [63]. Transmembrane domains were described using TMHMM 2.0 at http://www.cbs.dtu.dk/services/TMHMM-2.0/ [64], Phobius at http://phobius.sbc.su.se/ [65], SPLIT 4.0 at http://split.pmfst.hr/split/4/ [66] and SOSUI at http://bp.nuap.nagoya-u.ac.jp/sosui/sosui_submit.html [67]. The COILS program was used to predict coiled-coil structure [68] and PROSITE was used identify conserved motifs [69]. The progressiveMauve algorithm (version 2.3.1) [70] was used to create genome alignment of T5-like phages. CLUSTALW2.1 [71] at (http://www.ebi.ac.uk/Tools/msa/clustalw2/) was used to align amino acid sequences of AKFV33 with other T5-like phages. The nucleotide sequence of the AKFV33 was subsequently deposited with GenBank (Accession #: HQ665011).

### Analysis of structural proteins

CsCl-purified phage particles were separated by molecular weight on 10% Sodium dodecyl sulphate-polyacrylamide gel (Bio-Rad Laboratories, Mississauga, ON, Canada). Proteins were stained with Coomassie brilliant blue R250 (Bio-Rad Laboratories, Mississauga, ON, Canada) and subsequently characterized using Bionumerics software (Applied Maths, Austin, TX, USA). Bands of interest were excised, de-stained and dried using a SpeedVac (Savant Instrument, Farmingdale, NY, USA). Following reduction with 10 mM dithiothreitol (DTT) and alkylation with 55 mM iodoacetamide, the protein was digested with 20 ng of sequencing grade trypsin (Roche Diagnostics, Indianapolis, IN, USA) in 25 mM NH_4_HCO_3_ (pH 7.6) and incubated at 37°C overnight. The proteolytic peptides were then extracted, and further purified by a C18 Ziptip (Millipore, Billerica, MA, USA). The purified peptides were analyzed by matrix-assisted laser desorption ionization (MALDI) mass spectrometry using an QSTAR XL quadrupole time-of-flight (QqTOF) instrument (AB Sciex, Foster City, CA, USA) under a nitrogen laser (337 nm), with 2,5-dihydroxybenzoic acid as the matrix. Following the MALDI MS analysis, the peptide ions were sequenced by tandem mass spectrometry (MS/MS) using argon as the collision gas. Protein identification was achieved by Mascot (Matrix Science, London, UK) search against the National Centre for Biotechnology Information (NCBI) database, as well as the in-house protein database derived from AKFV33 genome sequences.

## Supporting Information

Figure S1 **Sequence logos representation of AKFV33 promoters.** Consensus sequences were plotted with WebLogo. Height of letter indicates degree of conservation.
(TIF)

Figure S2 **Ratio of phage AKFV33 codon usage relative to that of STEC O157:H7 EDL933.** The grey bars indicate that codons corresponding to phage-encoded tRNAs.
(TIF)

Figure S3 **Sequence comparison from the flap endonuclease (D15) to the conserved hypothetical protein (ORF141) among T5-like phages using a progressiveMAUVE algorithm.** The degree of sequence similarity is indicated by the intensity of the red region. The contiguous black boxes under the red region represent the position of the genes and the coloured boxes indicate the conserved genes. Lines link blocks with homology between two genomes. A, AKFV33; B, EPS7; C, H8; D, SPC35; E, T5 (ATCC11303-B5, GenBank Accession#: AY587007).
(TIF)

Figure S4 A, Phylogenetic characterization of the receptor binding protein (*pb5*) of T5-like phages. The sequences were subjected to “one click” phylogenetic analysis incorporating the Gblocks program to eliminate poorly aligned positions and divergent regions at phylogeny.fr [72]. Scale bar represents 0.6 substitutions; B, Amino acid sequence comparison of receptor binding protein (*pb5*) of T5-like phages. The sequence alignment was generated using CLUSTALW2.1. Residues are indicated with an asterisk (*) if identical, a colon (:) if conserved, and a period (.) if related.
(TIF)

Figure S5 **Amino acid sequence comparison of pore-forming tail tip protein (** ***pb2*** **) in phages AKFV33 and T5 (ATCC11303-B5).** The sequence alignment was generated using CLUSTALW2.1. Residues are indicated with an asterisk (*) if identical, a colon (:) if conserved, and a period (.) if related.
(TIF)

Table S1 **Feature of phage AKFV33 gene products and their functional assignments.**
(DOCX)

Table S2 **MALDI QqTOF MS and MS/MS analysis of phage AKFV33 structural proteins^a^.**
(DOCX)
