# Supplementary figures and images for: Genomic, Proteomic and Physiological Characterization of a T5-like Bacteriophage for Control of Shiga Toxin-Producing Escherichia coli O157:H7
Source: PLoS One. 2012 Apr 13;7(4):e34585. doi: 10.1371/journal.pone.0034585 (PMC3326045; doi:10.1371/journal.pone.0034585)

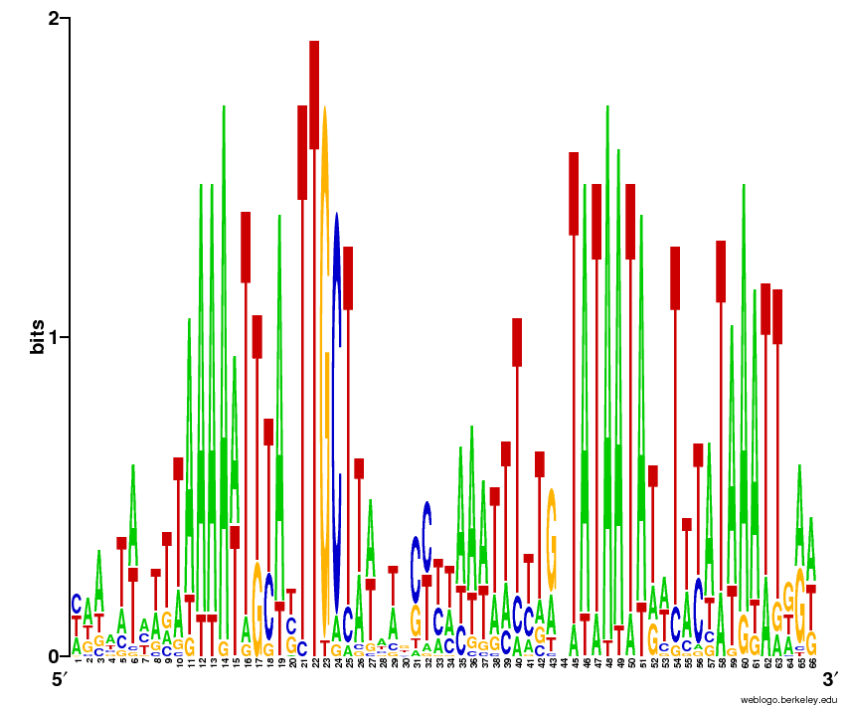

Supplement: Figure S1 — Sequence logos representation of AKFV33 promoters. Consensus sequences were plotted with WebLogo. Height of letter indicates degree of conservation. (TIF) [file pone.0034585.s001.tif]

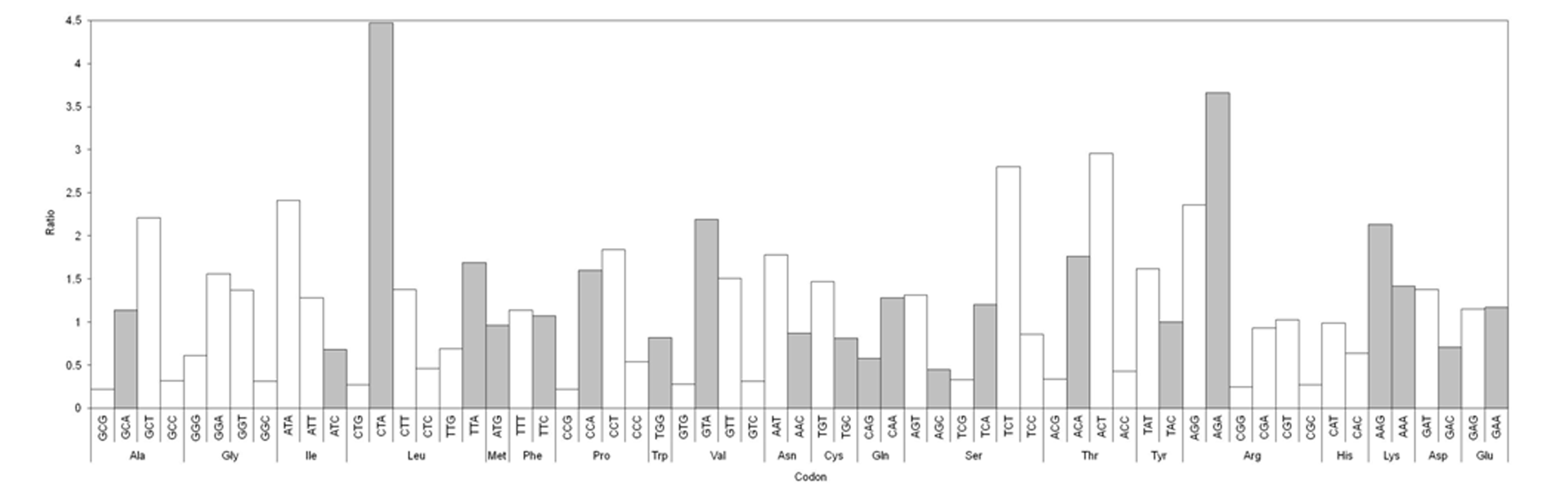

Supplement: Figure S2 — Ratio of phage AKFV33 codon usage relative to that of STEC O157:H7 EDL933. The grey bars indicate that codons corresponding to phage-encoded tRNAs. (TIF) [file pone.0034585.s002.tif]

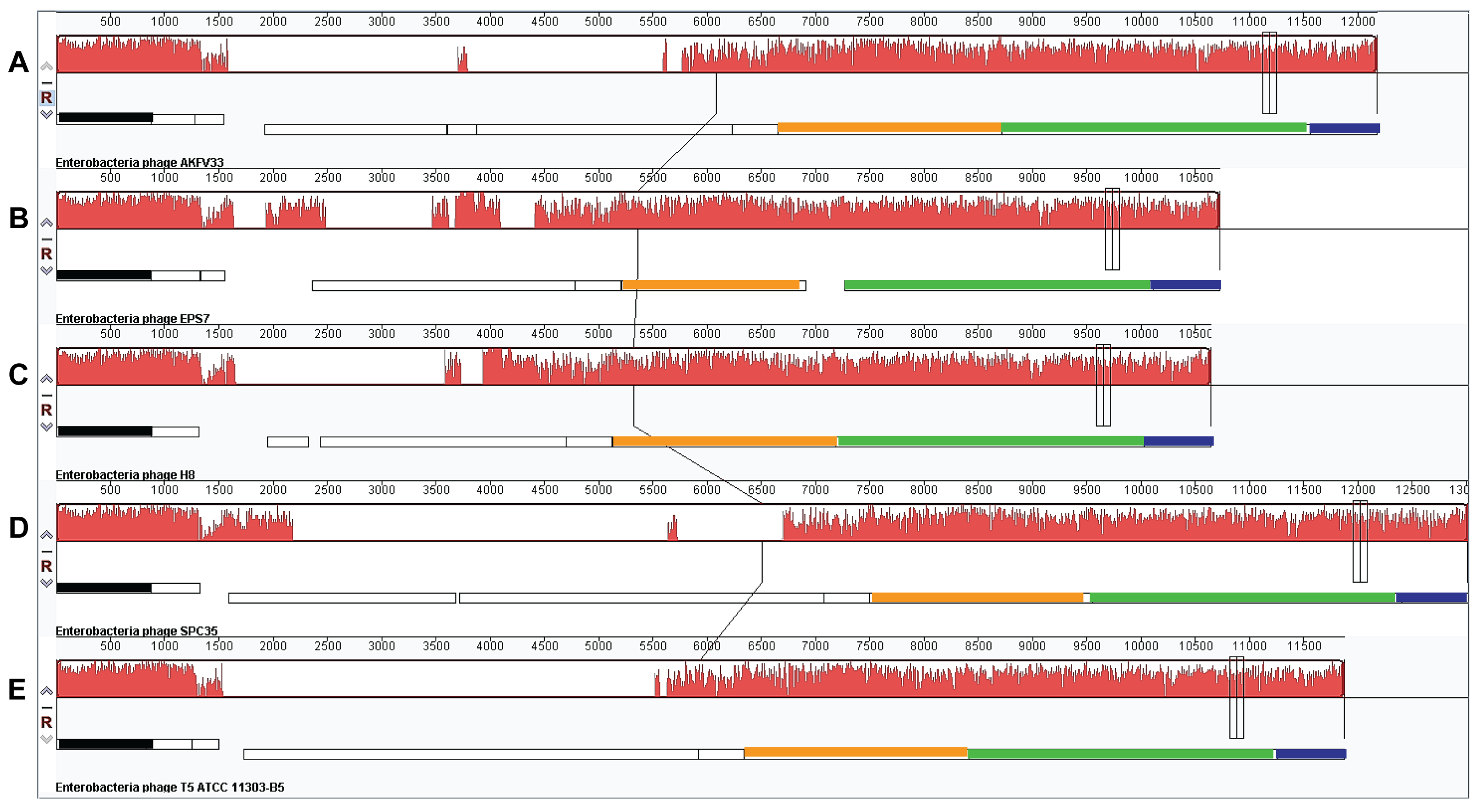

Supplement: Figure S3 — Sequence comparison from the flap endonuclease (D15) to the conserved hypothetical protein (ORF141) among T5-like phages using a progressiveMAUVE algorithm. The degree of sequence similarity is indicated by the intensity of the red region. The contiguous black boxes under the red region represent the position of the genes and the coloured boxes indicate the conserved genes. Lines link blocks with homology between two genomes. A, AKFV33; B, EPS7; C, H8; D, SPC35; E, T5 (ATCC11303-B5, GenBank Accession#: AY587007). (TIF) [file pone.0034585.s003.tif]

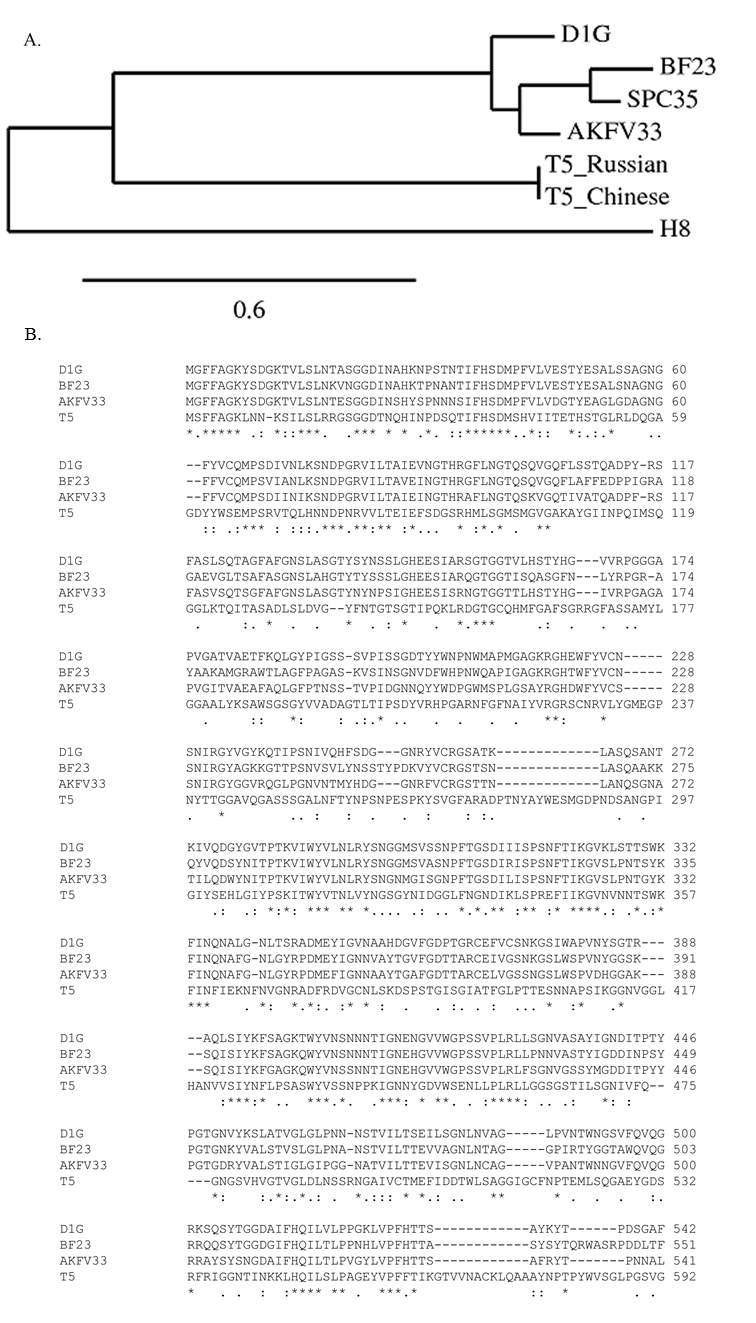

Supplement: Figure S4 — A, Phylogenetic characterization of the receptor binding protein (pb5) of T5-like phages. The sequences were subjected to “one click” phylogenetic analysis incorporating the Gblocks program to eliminate poorly aligned positions and divergent regions at phylogeny.fr [72]. Scale bar represents 0.6 substitutions; B, Amino acid sequence comparison of receptor binding protein (pb5) of T5-like phages. The sequence alignment was generated using CLUSTALW2.1. Residues are indicated with an asterisk (*) if identical, a colon (:) if conserved, and a period (.) if related. (TIF) [file pone.0034585.s004.tif]

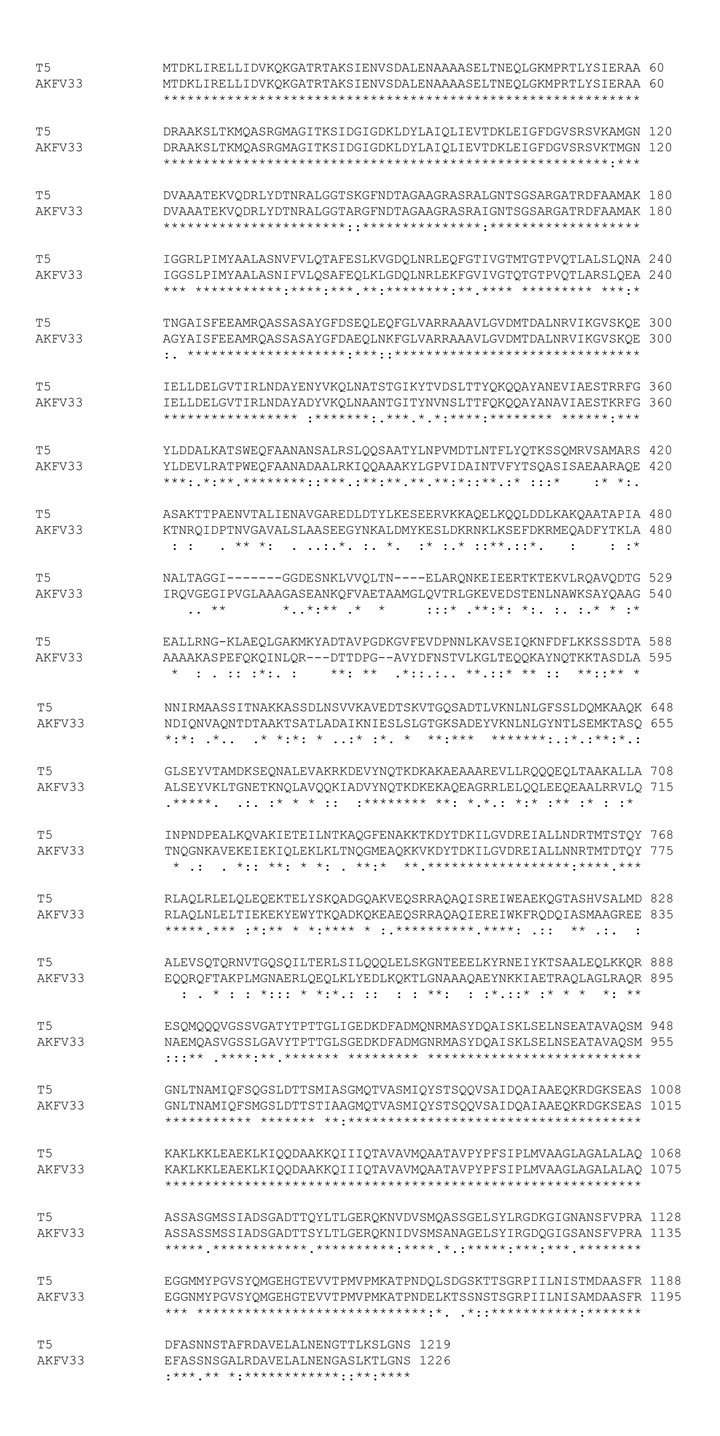

Supplement: Figure S5 — Amino acid sequence comparison of pore-forming tail tip protein ( pb2 ) in phages AKFV33 and T5 (ATCC11303-B5). The sequence alignment was generated using CLUSTALW2.1. Residues are indicated with an asterisk (*) if identical, a colon (:) if conserved, and a period (.) if related. (TIF) [file pone.0034585.s005.tif]
