# Supplementary material for: Genomic, Proteomic and Physiological Characterization of a T5-like Bacteriophage for Control of Shiga Toxin-Producing Escherichia coli O157:H7
Source: PLoS One. 2012 Apr 13;7(4):e34585. doi: 10.1371/journal.pone.0034585 (PMC3326045; doi:10.1371/journal.pone.0034585)
Supplement: Table S1 — Feature of phage AKFV33 gene products and their functional assignments. (DOCX) [file pone.0034585.s006.docx]

| **Table S1 Feature of phage AKFV33 gene products and their functional assignments** | | | | | | | | |
| --- | --- | --- | --- | --- | --- | --- | --- | --- |
|  | | | | | | | | |
| **ORF** | **Strand** | **Start−stop** | **Size (aa)/**  **MW (kDa)/pI** | **Motifs (n)** | **Function** | **Homologues** | **% ID^a^** | **E*-*value** |
| 1 *dmp* | − | 121−855 | 244/28.4/5.8 |  | deoxynucleoside-5'-monophosphatase | deoxynucleoside-5'-monophosphatase (phage T5, YP_006829.1) | 225/244 (92%) | 1E-129 |
| 2 | − | 942−1343 | 133/14.4/5.4 |  | conserved hypothetical protein | hypothetical protein T5.002 (phage T5, YP_006830.1) | 124/131 (94%) | 5E-64 |
| 3 | − | 1380−1637 | 85/9.6/4.1 |  | hypothetical protein |  |  | 5E-313 |
| 4 *A1* | − | 1695−3359 | 554/61.1/6.1 |  | phage DNA transfer protein | A1 (phage T5, YP_006832.1) | 514/556 (92%) | 0 |
| 5 | − | 3450−3647 | 65/7.2/9.1 | transmembrane domains (2) | conserved hypothetical protein | hypothetical protein T5.005 (phage T5, YP_006833.1) | 63/65 (96%) | 3E-28 |
| 6 *A2* | − | 3739−4146 | 135/14.3/8.8 |  | DNA-binding protein | A2 (phage T5, YP_006834.1) | 134/135 (99%) | 9E-68 |
| 7 | − | 4248−4499 | 83/9.1/9.1 |  | conserved hypothetical protein | hypothetical protein T5.007 (phage T5, YP_006835.1) | 66/82 (80%) | 5E-28 |
| 8 | − | 4733−4936 | 67/7.9/10.5 |  | conserved hypothetical protein | hypothetical protein T5.008 (phage T5, YP_006836.1) | 58/67 (86%) | 2E-25 |
| 9 | − | 4933−5223 | 96/11.0/10.7 |  | conserved hypothetical protein | hypothetical protein T5.009 (phage T5, YP_006837.1) | 33/35 (94%) | 1E-10 |
| 10 | + | 6054−7046 | 330/36.7/4.7 |  | conserved hypothetical protein | hypothetical protein T5.011 (Phage T5, YP_006839.1 ) | 226/336 (67%) | 1E-121 |
| 11 | + | 7163−7393 | 76/8.6/6.8 |  | conserved hypothetical protein | hypothetical protein T5.012 (phage T5, YP_006840.1 ) | 63/76 (82%) | 7E-28 |
| 12 | + | 7443−7592 | 49/5.0/8.0 | transmembrane domains (1) | conserved hypothetical protein | hypothetical protein AGC_0011 (phage EPS7, YP_001836934.1 ) | 23/26 (88%) | 1E-04 |
| 13 | + | 7595−7807 | 70/8.1/10.2 |  | conserved hypothetical protein | hypothetical protein T5.014 (phage T5, YP_006842.1) | 68/70 (97%) | 3E-31 |
| 14 | + | 7804−7947 | 47/5.0/8.3 | transmembrane domains (2) | hypothetical protein |  |  | 5E-313 |
| 15 | + | 8035−8394 | 119/13.4/6.6 |  | conserved hypothetical protein | hypothetical protein T5.015 (phage T5, YP_006843.1) | 104/114 (91%) | 8E-53 |
| 16 | + | 8423−8617 | 64/7.6/4.8 |  | conserved hypothetical protein | hypothetical protein T5.017 (phage T5, YP_006845.1) | 56/64 (87%) | 4E-26 |
| 17 | + | 9458−9595 | 45/5.4/6.5 |  | hypothetical protein |  |  | 5E-313 |
| 18 | − | 10282−11172 | 296/34.4/6.2 |  | conserved hypothetical protein | hypothetical protein T5.018 (phage T5, YP_006846.1) | 79/185 (42%) | 2E-31 |
| 19 | − | 11172−11771 | 199/21.9/5.8 |  | conserved hypothetical protein | hypothetical protein T5.019 (phage T5, YP_006847.1) | 147/200 (73%) | 4E-80 |
| 20 | − | 11771−11956 | 61/7.1/7.9 |  | conserved hypothetical protein | C-terminus of hypothetical protein T5.020 (phage T5, YP_006848.1) | 27/28 (96%) | 1E-07 |
| 21 | − | 11956−12189 | 77/8.8/9.2 | transmembrane domains (1) | conserved hypothetical protein | from amino acid of hypothetical protein T5.021 (phage T5, YP_006849.1 ) | 54/59 (91%) | 4E-22 |
| 22 | − | 12177−12467 | 96/11.3/4.6 |  | conserved hypothetical protein | hypothetical protein T5.022 (phage T5, YP_006850.1) | 72/96 (75%) | 1E-36 |
| 23 | − | 12460−12906 | 148/17.5/8.6 |  | conserved hypothetical protein | hypothetical protein T5.023 (phage T5, YP_006851.1) | 143/148 (96%) | 1E-82 |
| 24 | − | 12908−13111 | 67/7.8/4.3 |  | conserved hypothetical protein | hypothetical protein T5.024 (phage T5, YP_006852.1) | 62/67 (92%) | 2E-30 |
| 25 | − | 13166−13504 | 112/12.4/4.4 |  | conserved hypothetical protein | hypothetical protein T5.025 (phage T5, YP_006853.1) | 104/112 (92%) | 8E-51 |
| 26 | − | 13485−13946 | 153/18.1/5.9 |  | conserved hypothetical protein | hypothetical protein T5.026 (phage T5, YP_006854.1) | 150/153 (98%) | 1E-86 |
| 27 | − | 13943−14143 | 66/7.7/9.2 | transmembrane domains (1) | conserved hypothetical protein | hypothetical protein T5.027 (phage T5, YP_006855.1) | 63/66 (95%) | 3E-28 |
| 28 | − | 14243−14569 | 108/12.5/4.8 |  | conserved hypothetical protein | hypothetical protein T5.028 (phage T5, YP_006856.1) | 91/108 (84%) | 3E-46 |
| 29 | − | 14559−14804 | 81/9.2/7.9 | transmembrane domains (2) | conserved hypothetical protein | hypothetical protein T5.029 (phage T5, YP_006857.1) | 73/80 (91%) | 5E-35 |
| 30 | − | 14801−15082 | 93/10.4/8.9 | transmembrane domains (1) | conserved hypothetical protein | hypothetical protein T5.030 (phage T5, YP_006858.1) | 71/93 (76%) | 6E-30 |
| 31 | − | 15246−15497 | 83/9.5/9.4 |  | conserved hypothetical protein | hypothetical protein T5.032 (phage T5, YP_006860.1) | 82/83 (98%) | 8E-38 |
| 32 | − | 15576−16007 | 143/16.5/4.3 |  | conserved hypothetical protein | hypothetical protein T5.033 (phage T5, YP_006861.1) | 142/143 (99%) | 9E-79 |
| 33 | − | 16175−16765 | 196/22.1/8.5 | metallophosphatase (MPP) superfamily | putative serine/threonine protein phosphatase | putative serine/threonine protein phosphatase (phage EPS7, YP_001836958.1) | 157/171 (91%) | 5E-87 |
| 34 | − | 16755−17054 | 99/11.4/4.8 |  | hypothetical protein |  |  | 5E-313 |
| 35 | − | 17054−17917 | 286/32.6/5.3 | MPP superfamily | putative serine/threonine protein phosphatase | putative serine/threonine protein phosphatase (phage T5, YP_006863.1) | 261/286 (92%) | 2E-155 |
| 36 | − | 17920−18165 | 81/9.4/9.8 |  | conserved hypothetical protein | hypothetical protein T5.036 (phage T5, YP_006864.1 ) | 61/81 (75%) | 7E-25 |
| 37 | − | 18170−18547 | 125/14.6/4.3 |  | conserved hypothetical protein | hypothetical protein AGC_0039 (phage EPS7, YP_001836962.1) | 68/102 (66%) | 3E-34 |
| 38 *nrdC* | − | 18730−19020 | 96/11.1/5.9 | thioredoxin (TRX) | putative thioredoxin | putative thioredoxin (phage T5, YP_006865.1) | 95/96 (98%) | 2E-49 |
| 39 | − | 19013−19444 | 143/16.4/8.8 |  | conserved hypothetical protein | hypothetical protein T5.038 (phage T5, YP_006866.1) | 128/143 (89%) | 2E-64 |
| 40 | − | 19520−19936 | 138/15.7/8.7 |  | conserved hypothetical protein | hypothetical protein T5.039 (phage T5, YP_006867.1 ) | 132/138 (95%) | 1E-70 |
| 41 *lys* | − | 20014−20427 | 137/15.3/8.0 |  | lysozyme | lysozyme (phage T5, YP_006868.1) | 136/137 (99%) | 5E-74 |
| 42 *C1* | − | 20424−21080 | 218/24.7/8.4 | transmembrane domains (1) | putative holin | putative holin (phage T5, YP_006869.1) | 208/218 (95%) | 1E-114 |
| 43 | − | 21237−21833 | 198/22.8/4.9 | crotonase-like superfamily | putative ATP-dependent Clp protease | putative ATP-dependent Clp protease (phage T5, YP_006870.1) | 194/196 (98%) | 1E-111 |
| 44 *dnk* | − | 21846−22598 | 250/28.7/4.8 | deoxynucleoside monophosphate kinase | deoxynucleoside-5'-monosphate kinase | deoxynucleoside-5'- monophosphate kinase (phage T5, YP_006871.1) | 248/250 (99%) | 1E-142 |
| 45 | − | 22598−22951 | 124/13.5/6.1 |  | conserved hypothetical protein | hypothetical protein T5.044 (phage T5, YP_006872.1) | 113/117 (96%) | 2E-63 |
| 46 | − | 22882−23331 | 149/16.5/9.2 | transmembrane domains (1) | conserved hypothetical protein | hypothetical protein AGC_0048 (phage EPS7, YP_001836971.1) | 130/149 (87%) | 1E-65 |
| 47 | − | 23288−23986 | 232/27.3/6.0 |  | conserved hypothetical protein | hypothetical protein T5.047 (phage T5, YP_006875.1) | 220/232 (94%) | 1E-126 |
| 48 | − | 24141−24485 | 114/13.2/9.4 |  | conserved hypothetical protein | hypothetical protein T5.048 (phage T5, YP_006876.1) | 86/114 (75%) | 1E-45 |
| 49 | − | 24596−24880 | 94/10.9/9.6 | transmembrane domains (2) | conserved hypothetical protein | hypothetical protein T5.049 (phage T5, YP_006877.1) | 76/93 (81%) | 4E-36 |
| 50 | − | 24877−25173 | 98/11.4/8.0 | transmembrane domains (1) | conserved hypothetical protein | hypothetical protein T5.050 (phage T5, YP_006878.1) | 90/98 (91%) | 3E-46 |
| 51 | − | 25151−25546 | 131/15.2/6.3 |  | conserved hypothetical protein | hypothetical protein AGC_0053 (phage EPS7, YP_001836976.1) | 102/115 (88%) | 8E-54 |
| 52 | − | 25539−25838 | 99/11.0/8.6 |  | conserved hypothetical protein | hypothetical protein AGC_0054 (phage EPS7, YP_001836977.1) | 88/99 (88%) | 5E-45 |
| 53 | − | 25831−26112 | 93/10.6/4.4 |  | conserved hypothetical protein | hypothetical protein T5.053 (phage T5, YP_006881.1) | 83/93 (89%) | 3E-45 |
| 54 | − | 26189−26536 | 115/14.4/9.3 |  | conserved hypothetical protein | hypothetical protein T5.054 (phage T5, YP_006882.1) | 102/115 (88%) | 4E-60 |
| 55 | − | 26658−26972 | 104/12.1/9.1 |  | conserved hypothetical protein | hypothetical protein T5.055 (phage T5, YP 006883.1) | 80/104 (76%) | 3E-40 |
|  |  |  |  |  |  | hypothetical protein (phage BF23, CAE53182.1) | 86/104 (82%) | 3E-45 |
| 56 | − | 27054−27422 | 122/13.9/5.6 | ribonucleotide reductase and Pyruvate formate lyase (RNP_PFL) | putative acetyltransferase-related protein | putative acetyltransferase-related protein (phage T5, YP_006884.1) | 115/122 (94%) | 2E-39 |
| 57 | − | 27608−27802 | 64/7.1/9.6 |  | conserved hypothetical protein | hypothetical protein HMPREF9540_02224 (*E  coli* MS 115-1, ZP_07135031.1) | 36/60 (60%) | 9E-13 |
| 58 | − | 28090−28269 | 59/6.6/10.2 |  | conserved hypothetical protein | hypothetical protein AGC_0061 (phage EPS7, YP_001836984.1) | 56/69 (94%) | 5E-26 |
|  |  |  |  |  |  | hypothetical protein (*Salmonella* phage 5, CAE53211.1) | 59/59 (100%) | 4E-27 |
| 59 | − | 28296−28589 | 97/11.2/9.5 | motif of ribosomal proteins (KOW superfamily ) | conserved hypothetical protein | hypothetical protein T5.058 (phage T5, YP_006886.1) | 78/96 (81%) | 5E-38 |
|  |  |  |  |  |  | hypothetical protein (*Salmonella* phage 5, CAE53212.1) | 92/97 (94%) | 2E-47 |
| 60 | − | 28748−28912 | 54/6.3/6.2 |  | conserved hypothetical protein | hypothetical protein T5.059 (phage T5, YP_006887.1) | 43/54 (79%) | 3E-17 |
|  |  |  |  |  |  | hypothetical protein (*Salmonella* phage 5, CAE53214.1) | 54/54 (100%) | 8E-22 |
| 61 | − | 28929−29126 | 65/7.7/5.0 |  | conserved hypothetical protein | hypothetical protein T5.060 (phage T5, YP_006888.1) | 54/61 (88%) | 1E-23 |
|  |  |  |  |  |  | hypothetical protein AGC_0064 (phage EPS7, YP_001836987.1) | 50/65 (76%) | 1E-23 |
|  |  |  |  |  |  | hypothetical protein (*Salmonella* phage 5, CAE53215.1) | 64/65 (98%) | 5E-31 |
| 62 | − | 29397−29591 | 64/7.0/4.4 |  | conserved hypothetical protein | hypothetical protein T5.062 (phage T5, YP_006890.1) | 58/64 (90%) | 6E-28 |
| 63 | − | 30789−31142 | 117/13.0/4.2 |  | conserved hypothetical protein | hypothetical protein AGC_0071 (phage EPS7, YP_001836994.1) | 108/117 (96%) | 7E-58 |
| 64 | − | 31326−31493 | 55/6.5/9.6 |  | conserved hypothetical protein | hypothetical protein T5.065 (phage T5, YP_006893.1) | 50/55 (90%) | 1E-20 |
| 65 | − | 32292−32639 | 115/13.3/4.3 |  | conserved hypothetical protein | hypothetical protein T5.067 (phage T5, YP_006895.1) | 113/115 (98%) | 2E-63 |
| 66 | − | 32743−32901 | 52/6.0/4.7 |  | hypothetical protein |  |  | 5E-313 |
| 67 | − | 33005−33190 | 61/7.2/6.7 |  | conserved hypothetical protein | hypothetical protein T5.068 (phage T5, YP_006896.1) | 61/61 (100%) | 2E-25 |
| 68 | − | 33459−33647 | 62/7.1/6.0 |  | conserved hypothetical protein | hypothetical protein AGC_0075 (phage EPS7, YP_001836998.1) | 28/49 (57%) | 1E-10 |
| 69 | − | 33644−33868 | 74/8.4/4.8 |  | conserved hypothetical protein | hypothetical protein AGC_0076 (phage EPS7, YP_001836999.1) | 64/74 (86%) | 1E-28 |
| 70 | − | 33919−34191 | 90/10.8/9.8 |  | conserved hypothetical protein | hypothetical protein T5.071 (phage T5, YP_006899.1) | 71/90 (78%) | 2E-35 |
|  |  |  |  |  |  | hypothetical protein (phage BF23, CAE53198.1) | 75/90 (83%) | 1E-37 |
| 71 | − | 34678−34953 | 91/10.2/4.2 |  | conserved hypothetical protein | hypothetical protein T5.072 (phage T5, YP_006900.1) | 86/91 (94%) | 2E-44 |
| 72 | − | 35044−35250 | 68/7.4/4.6 |  | conserved hypothetical protein | hypothetical protein AGC_0079 (phage EPS7, YP_001837002.1) | 56/68 (82%) | 4E-23 |
| 73 | − | 35243−35410 | 55/6.5/7.9 |  | conserved hypothetical protein | hypothetical protein T5.074 (phage T5, YP_006902.1) | 42/55 (76%) | 8E-16 |
| 74 | − | 35604−35789 | 61/6.6/4.1 | chaperone protein DnaJ | conserved hypothetical protein | hypothetical protein AGC_0083 (phage EPS7, YP_001837006.1) | 37/61 (60%) | 5E-16 |
| 75 | − | 35799−35987 | 62/7.4/4.6 |  | conserved hypothetical protein | Sequence similarity at C-terminus to hypothetical protein (*Salmonella* phage 5, CAE53227.1) | 41/42 (97%) | 2E-15 |
| 76 | − | 36202−36720 | 172/19.0/6.9 |  | conserved hypothetical protein | hypothetical protein T5.076 (phage T5, YP_006904.1) | 151/172 (87%) | 2E-84 |
| 77 | − | 36817−37764 | 315/35.3/8.8 | flotillin like proteins (Band_7 superfamily); transmembrane domains (1) | conserved hypothetical protein | hypothetical protein T5.080 (phage T5, YP_006908.1 ) | 252/315 (80%) | 1E-133 |
| 78 | − | 38272−38409 | 45/4.9/6.0 |  | hypothetical protein |  |  | 5E-313 |
| 79 | − | 38562−38996 | 144/16.4/9.1 |  | conserved hypothetical protein | hypothetical protein T5.081 (phage T5, YP_006909.1) | 87/144 (60%) | 9E-39 |
| 80 | − | 38996−39166 | 56/6.1/10.4 |  | conserved hypothetical protein | hypothetical protein AGC_0090 (phage EPS7, YP_001837013.1) | 54/56 (96%) | 6E-22 |
| 81 *SleB* | − | 39235−39684 | 149/16.9/9.7 | cell wall hydrolase (Hydrolase_2 superfamily) | cell wall hydrolase SleB | hypothetical protein T5.083 (phage T5, YP_006911.1) | 125/149 (83%) | 3E-65 |
|  |  |  |  |  |  | spore cortex-lytic enzyme precursor (Phage T5, AAX12015.1) | 124/149 (83%) | 2E-64 |
| 82 | − | 39690−40007 | 105/12.2/4.9 |  | conserved hypothetical protein | hypothetical protein T5.084 (phage T5, YP_006912.1) | 101/105 (96%) | 1E-53 |
| 83 | − | 40450−41088 | 212/24.0/6.8 |  | conserved hypothetical protein | hypothetical protein T5.085 (phage T5, YP_006913.1) | 209/212 (98%) | 1E-107 |
| 84 | − | 41142−41324 | 60/6.8/11.6 |  | conserved hypothetical protein | hypothetical protein T5.086 (phage T5, YP_006914.1) | 58/60 (96%) | 1E-24 |
| 85 | − | 41395−42096 | 233/25.9//5.8 |  | putative metallopeptidase | putative metallopeptidase (phage T5, YP_006915.1) | 221/233 (94%) | 1E-128 |
| 86 | − | 42125−42361 | 78/9.3/4.6 |  | conserved hypothetical protein | hypothetical protein T5.088 (phage T5, YP_006916.1) | 73/78 (93%) | 2E-37 |
| 87 | − | 42403−42618 | 71/8.0/6.2 |  | conserved hypothetical protein | hypothetical protein AGC_0098 (phage EPS7, YP_001837021.1 ) | 69/71 (97%) | 8E-29 |
| 88 | − | 42682−43197 | 171/19.1/10.6 |  | conserved hypothetical protein | hypothetical protein T5.089 (phage T5, YP_006917.1) | 130/156 (83%) | 2E-68 |
| 89 | − | 43281−43559 | 92/10.7/9.7 |  | conserved hypothetical protein | hypothetical protein T5.090 (phage T5, YP_006918.1) | 91/92 (98%) | 1E-45 |
| 90 *rnh* | − | 43636−44112 | 158/17.9/5.4 | RNaseH | RNaseH | putative RNaseH ribonuclease (phage T5, YP_006919.1) | 153/158 (96%) | 3E-89 |
| 91 | − | 44112−44375 | 87/10.3/6.7 |  | hypothetical protein |  |  | 5E-313 |
| 92 | − | 44470−44823 | 117/13.8/9.1 | DUF3307 (unknown function)/  transmembrane domains (2) | conserved hypothetical protein | hypothetical protein AGC_0104 (phage EPS7, YP_001837027.1) | 79/87 (90%) | 4E-40 |
| 93 *td* | − | 44827−45681 | 284/32.6/6.3 | thymidylate synthase | thymidylate synthase | (phage EPS7, YP_001837028.1) | 246/284 (86%) | 1E-142 |
| 94 *frd* | − | 45681−46214 | 177/19.8/4.5 | dihydrofolate reductase | putative dihydrofolate reductase | putative dihydrofolate reductase (phage T5, YP_006921.1) | 166/177 (93%) | 3E-90 |
| 95 *nrdB* | − | 46211−47356 | 381/43.6/4.7 | ribonucleotide reductase | putative aerobic ribonucleoside diphosphate reductase, small subunit | putative aerobic ribonucleoside diphosphate reductase, small subunit (phage T5, YP_006922.1) | 376/381 (98%) | 0 |
| 96 *hegH* | − | 47353−47850 | 165/18.9/9.1 | putative NHN endonuclease | putative homing endonuclease | putative H-N-H-endonuclease P-TflV III (phage T5, YP_006923.1) | 68/161 (42%) | 5E-27 |
| 97 *nrdA* | − | 47932−50367 | 811/91.8/5.8 | ribonucleotide reductase | putative aerobic ribonucleoside diphosphate reductase, large subunit | putative aerobic ribonucleoside diphosphate reductase, large subunit (phage T5, YP_006924.1 ) | 762/789 (96%) | 0 |
| 98 | − | 50405−50626 | 73/8.2/9.9 |  | conserved hypothetical protein | hypothetical protein AGC_0110 (phage EPS7, YP_001837033.1) | 60/73 (82%) | 3E-27 |
| 99 *phoH* | − | 50628−51380 | 250/28.0/6.8 | PhoH | phosphate starvation-inducible protein | putative PhoH-like protein (phage T5, YP_006926.1) | 247/250 (98%) | 1E-139 |
| 100 | + | 51735−52403 | 222/25.2/8.8 | DNA-binding protein (T5ORF172) | conserved hypothetical protein | hypothetical protein T5.154 (phage T5, YP_006982.1) | 82/114 (38%) | 2E-37 |
| 101 *nrdD* | + | 52396−54243 | 615/6.9/5.7 | class III ribonucleotide reductase (RNR_III) | anaerobic ribonucleoside triphosphate reductase | anaerobic ribonucleoside triphosphate reductase (phage EPS7, YP_001837035.1 ) | 562/610 (92%) | 0 |
| 102 | + | 54344−54724 | 126/14.2/4.1 |  | conserved hypothetical protein | hypothetical protein T5.100 (phage T5, YP_006928.1 ) | 122/126 (96%) | 3E-67 |
| 103 | + | 54717−54923 | 68/8.0/6.7 |  | conserved hypothetical protein | hypothetical protein T5.101 (phage T5, YP_006929.1) | 62/67 (92%) | 5E-27 |
| 104 | + | 54892−55086 | 64/7.4/5.2 |  | conserved hypothetical protein | hypothetical protein AGC_0115 (phage EPS7, YP_001837038.1) | 56/64 (87%) | 1E-24 |
| 105 | + | 55086−55904 | 272/31.2/5.6 | Sir2 superfamily | Sir2-like protein | putative Sir2-like protein (phage T5, YP_006930.1) | 249/267 (93%) | 1E-147 |
| 106 | + | 55882−56064 | 60/7.3/3.9 |  | conserved hypothetical protein | hypothetical protein AGC_0118(phage EPS7, YP_001837041.1) | 55/55 (100%) | 1E-28 |
| 107 | + | 56045−56230 | 61/7.0/9.7 | transmembrane domains (2) | conserved hypothetical protein | hypothetical protein AGC_0119(phage EPS7, YP_001837042.1) | 40/40 (100%) | 1E-16 |
| 108 | + | 56217−56723 | 168/19.0/5.7 |  | conserved hypothetical protein | hypothetical protein T5.105 (phage T5, YP_006933.1) | 152/168 (90%) | 6E-83 |
| 109 | + | 56726−57154 | 142/16.4/4.7 |  | conserved hypothetical protein | hypothetical protein T5.106 (phage T5, YP_006934.1) | 131/142 (92%) | 1E-73 |
| 110 | + | 57164−57556 | 130/15.0/4.5 |  | conserved hypothetical protein | hypothetical protein T5.107 (phage T5, YP_006935.1) | 122/130 (93%) | 1E-68 |
| 111 *hegI* | + | 57696−58148 | 150/17.5/9.7 |  | putative homing endonuclease | predicted phage DNA Endonuclease (*Pseudomonas* phage Φ2, YP_003345491.1) | 50/154 (32%) | 1E-10 |
| 112 *obp* | + | 58682−61471 | 929/105.7/6.2 |  | putative replication origin binding protein | putative replication origin binding protein (phage T5, YP_006936.1) | 906/927 (97%) | 0 |
| 113 | + | 61455−61688 | 77/9.0/9.9 |  | conserved hypothetical protein | hypothetical protein T5.110 (phage T5, YP_006938.1) | 73/77 (94%) | 1E-34 |
| 114 *D2* | + | 61757−62461 | 234/26.7/6.3 |  | conserved hypothetical protein | D2 protein (phage T5, YP_006939.1) | 232/234 (99%) | 1E-130 |
| 115 | + | 62454−62705 | 83/9.2/4.0 |  | conserved hypothetical protein | hypothetical protein T5.112 (phage T5, YP_006940.1) | 76/83 (91%) | 6E-28 |
| 116 *D3* | + | 62809−63219 | 136/14.5/5.7 |  | conserved hypothetical protein | D3 protein (phage T5, YP_006941.1) | 102/136 (75%) | 8E-48 |
| 117 | + | 63256−63552 | 98/11.6/9.3 |  | conserved hypothetical protein | hypothetical protein T5.114 (phage T5, YP_006942.1) | 82/98 (83%) | 5E-38 |
| 118 | + | 63603−63911 | 102/12.0/4.6 | aspartate aminotransferase (AAT) | conserved hypothetical protein | hypothetical protein T5.115 (phage T5, YP_006943.1) | 102/102 (100%) | 4E-53 |
| 119 *ligA* | + | 63999−64970 | 323/36.4/5.0 | NAD-dependent DNA ligase | NAD-dependent DNA ligase subunit A | NAD-dependent DNA ligase subunit A (phage T5, YP_006944.1) | 320/323 (99%) | 0 |
| 120 *ligB* | + | 65173−65943 | 256/28.0/8.6 | NAD+dependent DNA ligase | NAD-dependent DNA ligase subunit B | NAD-dependent DNA ligase subunit B (phage T5, YP_006945.1) | 247/259 (95%) | 1E-136 |
| 121 *D5* | + | 65936−66703 | 255/27.1/6.8 | helix-turn-helix XRE-family like proteins (DNA binding proteins) | conserved hypothetical protein | D5 protein (phage T5, YP_006946.1) | 229/240 (95%) | 1E-120 |
| 122 *D6* | + | 66735−68258 | 507/57.6/5.4 |  | putative replicative DNA helicase | D6 protein (putative replicative DNA helicase (phage T5, YP_006947.1) | 472/507 (93%) | 0 |
| 123 *pri* | + | 68255−69145 | 296/33.0/6.6 | DNA replication primase | DNA replication primase | putative DNA replication primase (phage T5, YP_006949.1) | 295/296 (99%) | 1E-168 |
| 124 *pol* | + | 69208−71775 | 855/97.6/5.8 | DNA polymerase | DNA polymerase | DNA polymerase (phage T5, YP_006950.1) | 874/855 (99%) | 0 |
| 125 | + | 71768−72265 | 165/19.4/9.8 |  | conserved hypothetical protein | hypothetical protein T5.123 (phage T5, YP_006951.1) | 164/165 (99%) | 9E-90 |
| 126 *D10* | + | 72262−73614 | 450/50.3/8.7 | helicase | putative ATP-dependent helicase | putative ATP-dependent helicase (phage T5, YP_006952.1) | 445/450 (98%) | 0 |
| 127 | + | 73751−74119 | 122/14.2/8.8 |  | conserved hypothetical protein | hypothetical protein AGC_0138 (phage EPS7, YP_001837061.1) | 109/120 (90%) | 1E-57 |
| 128  *D11* | + | 74112−74885 | 257/28.9/5.0 |  | conserved hypothetical protein | D11 protein (phage T5, YP_006954.1) | 251/257 (97%) | 1E-141 |
| 129 *D12* | + | 74922−75899 | 325/37.3/5.9 | MPP superfamily | putative recombination endonuclease subunit D12 | putative recombination endonuclease subunit D12 (phage EPS7, YP_001837063.1) | 301/325 (92%) | 1E-179 |
| 130 *D13* | + | 75922−77718 | 598/67.0/5.3 | endonuclease | putative recombination endonuclease subunit D13 | putative recombination endonuclease subunit D13 (phage T5, YP_006956.1) | 556/598 (92%) | 0 |
| 131 *D14* | + | 77722−78204 | 160/18.5/6.2 | restriction  endonucleases  like superfamily | conserved hypothetical protein | D14 protein (phage T5, YP_006957.1) | 160/160 (100%) | 3E-91 |
| 132  *D15* | + | 78204−79079 | 291/33.5/5.2 | T5-5'nuclease | flap endonuclease | flap endonuclease (phage T5, YP_006958.1) | 286/291 (98%) | 1E-163 |
| 133 *dut* | + | 79076−79522 | 148/16.1/6.1 | deoxyUTP pyrophosphatase | deoxyUTP pyrophosphatase | deoxyUTP pyrophosphatase (phage T5, YP_006959.1) | 142/148 (95%) | 3E-77 |
| 134 | + | 79485−79748 | 87/9.8/4.4 |  | conserved hypothetical protein | hypothetical protein T5.132 (phage T5, YP_006960.1) | 69/87 (79%) | 1E-34 |
| 135 *ltpB* | − | 80124−81797 | 557/60.5/4.9 | coiled-coil structure (2) | tail fiber protein | Tail fiber (phage phiEco32, YP_001671760.1) | 200/534 (37%) | 2E-70 |
| 136 | − | 81808−82074 | 88/10.2/4.8 |  | conserved hypothetical protein | hypothetical protein ECP_1187 (*E. coli* 536, YP_669099.1) | 27/92 (29%) | 9E-05 |
|  |  |  |  |  |  | conserved hypothetical protein (*E. coli* MS 21-1, ZP_07152396.1) | 49/88 (55%) | 4E-26 |
| 137  *ltpA* | − | 82075−84429 | 784/83.1/5.0 | DUF1640 (unkown function protein domain derived from hypothetical eukaryotic proteins; coiled-coil structure (2) | putative tail fiber protein | conserved domain protein (*Escherichia coli* MS 196-1, ZP_07191982.1) | 189/454 (41%) | 4E-81; |
|  |  |  |  |  |  | putative side tail fiber protein homologue from prophage (*E. coli* IAI39, YP_002407901.1) | 175/448 (39%) | 8E-72 |
| 138 | − | 84429−84851 | 140/15.4/4.8 |  | putative phage tail protein | putative phage tail protein (phage EPS7, YP_001837070.1) | 114/140 (79%) | 1E-58 |
| 139 *pb4* | − | 84858−86915 | 685/74.9/4.9 |  | tail protein Pb4 | tail protein Pb4 (phage T5, YP_006965.1) | 525/555 (94%) | 0 |
| 140 *pb3* | − | 86915−89764 | 949/106.9/5.2 |  | tail protein Pb3 | tail protein Pb3 (phage EPS7, YP_001837072.1) | 820/949 (86%) | 0 |
| 141 | − | 89761−90375 | 204/22.7/5.5 |  | conserved hypothetical protein | hypothetical protein AGC_0150 (phage EPS7, YP_001837073.1) | 174/204 (85%) | 1E-101 |
| 142 *pb2* | − | 90485−94165 | 1226/132.3/5.9 | zinc metallopepidase; coilded-coil structure(7); transmembrane domain (2) | pore-forming tail tip protein | pore-forming tail tip protein (phage T5, YP_006968.1) | 731/1232 (59%) | 0 |
| 143 | − | 94249−94617 | 122/13.7/6.8 |  | conserved hypothetical protein | hypothetical protein T5.142 (phage T5, YP_006970.1) No ribosome-binding site | 109/122 (89%) | 7E-57 |
| 144 | − | 94679−95083 | 134/15.14.8 |  | conserved hypothetical protein | hypothetical protein T5.143 (phage T5, YP_006971.1) | 128/134 (95%) | 8E-69 |
| 145 | − | 95080−95979 | 299/34.3/5.1 |  | minor tail protein | putative tail protein (phage T5, YP_006972.1) | 212/298 (71%) | 1E-125 |
| 146 *N4* | − | 95984−97381 | 465/50.5/4.7 | bacterial Ig-like domain (Big_2 superfamily,host-cell interaction) | major tail protein | major tail protein (phage T5, YP_006973.1) | 414/465 (89%) | 0 |
| 147 | − | 97408−97893 | 161/18.3/4.9 |  | conserved hypothetical protein | hypothetical protein T5.146 (phage T5, YP_006974.1) | 159/161 (98%) | 2E-87 |
| 148 | − | 97897−98664 | 255/27.8/9.9 |  | conserved hypothetical protein | hypothetical protein AGC_0158 (phage EPS7, YP_001837081.1) | 232/255 (90%) | 1E-124 |
| 149 | − | 98664−99176 | 170/19.2/5.1 |  | conserved hypothetical protein | hypothetical protein T5.148 (phage T5, YP_006976.1) | 161/170 (94%) | 2E-89 |
| 150 | − | 99236−100612 | 458/50.6/5.1 | capsid | major head protein precursor | major head protein precursor (phage EPS7, YP_001837083.1) | 412/458 (89%) | 0 |
| 151 | − | 100630−101262 | 210/23.4/5.3 | caudovirus prohead protease (Peptidase_U35) | probable prohead protease | probable prohead protease (phage EPS7, YP_001837084.1) | 208/210 (99%) | 1E-115 |
| 152 | − | 101266−101748 | 160/16.8/8.9 | immunoglobulin domain (Ig) | putative tail protein | putative tail protein (phage T5, YP_006979.1) | 138/163 (84%) | 8E-70 |
| 153 | − | 101745−102962 | 405/45.3/5.7 | phage portal protein | portal protein | portal protein (phage T5, YP_006980.1) | 397/403 (98%) | 0 |
| 154 | − | 102962−103399 | 145/17.0/9.2 |  | conserved hypothetical protein | hypothetical protein T5.153 (phage T5, YP_006981.1) | 144/145 (99%) | 6E-80 |
| 155 | − | 103389−104063 | 224/25.6/8.4 | DNA-binding protein (T5ORF172) | conserved hypothetical protein | hypothetical protein YomD (phage T5, AAX12080.1) | 216/224 (96%) | 1E-126 |
|  |  |  |  |  |  | hypothetical protein T5.154 (phage T5, YP_006982.1) | 216/224 (96%) | 1E-126 |
| 156 | − | 104235−105551 | 438/49.7/5.8 | terminase | terminase, large subunit | terminase, large subunit (phage T5, YP_006983.1) | 437/438 (99%) | 0 |
| 157 *SciB* | − | 105551−106033 | 160/17.9/4.7 |  | SciB protein | hypothetical protein T5.156 (phage T5, YP_006984.1) | 128/160 (80%) | 4E-63 |
| 158 *pb5* | − | 106044−107801 | 585/62.5/7.7 |  | receptor-binding tail protein | receptor recognition protein (phage D1G, ACN72594.1) | 421/579 (72%) | 0 |
|  |  |  |  |  |  | receptor-binding protein (phage BF23, AAZ03642.1) | 396/596 (66%) | 0 |
|  |  |  |  |  |  | receptor-binding tail protein (phage T5, YP_006985.1) | 191/653 (29%) | 5E-50 |
| 159 *flp* | + | 107887−108153 | 88/9.9/9.5 |  | receptor-blocking protein | receptor-blocking protein (phage BF23, AAZ03643.1) | 88/88 (100%) | 3E-46 |
| 160 | + | 108331−108459 | 42/5.0/10.0 | transmembrane domains (1) | conserved hypothetical protein | hypothetical protein T5.159 (phage T5, YP_006987.1) | 24/42 (57%) | 6E-06 |

a, ID%, percent amino acid identity in region of BLASTP and PSI-BLAST alignment.
