# Supplementary material for: Genomic, Proteomic and Physiological Characterization of a T5-like Bacteriophage for Control of Shiga Toxin-Producing Escherichia coli O157:H7
Source: PLoS One. 2012 Apr 13;7(4):e34585. doi: 10.1371/journal.pone.0034585 (PMC3326045; doi:10.1371/journal.pone.0034585)
Supplement: Table S2 — MALDI QqTOF MS and MS/MS analysis of phage AKFV33 structural proteinsa. (DOCX) [file pone.0034585.s007.docx]

| **Table S2.** **MALDI QqTOF MS and MS/MS analysis of phage AKFV33 structural proteins^a^** | | | | |
| --- | --- | --- | --- | --- |
|  |  |  |  |  |
| **m/z**  **(Meas.)** | **[MH+]**  **(calc.)** | **Error**  **(Da)** | **Peptide** | **Sequence**^b^ |
| **Band A** |  | | | |
| **1** MTIDINKLKE ELGLGDLAKS LEGLTAAQKA AEAERMRKEQ EEKELARMND  **51** LVSKAVGEDR QKLEEALELV KSLDEKSKKS AELFAQTVEK QQETIVGLQD  **101** EIKSLLAARE GRSFVGDSVA KALYGTQEAF EDEVEKLVLL SYMMEKDVFE  **151** TEHGKAHVKA VNGSSSVSMS SEAYETIFST R**IIRDLQKEL VVGALFDELP**  **201 MSSKILTMLV EPEAGKATWV DASKFGTDET VGDEVKGTLT EISFK**TYK**LA**  **251 AKSFITDETE EDAIFSLLPL LR**KR**LIEAHA VSIEEAFMTG NGTGQPK**GLL  **301** TLASEDSAKV TTEAKADGSV LVTAKTISKL RRKLGRHGLK LSK**LVLIVSM**  **351 DAYYDLLEDE EWQDVAQVGN DAVKLQGQVG RIYGLPVVVS EYFPAKAADK**  **401 EFAVIVYKDN FVMPR**QR**AVT VERER**QAGK**Q RDAYYVTQRV NLQR**YFSNGV  **451** VSGAYAAS | | | | |
| 629.375 | 629.373 | 0.002 | 440-444 | (R)VNLQR(Y) |
| 757.424 | 757.432 | -0.008 | 375-381 | (K)LQGQVGR(I) |
| 959.531 | 959.527 | 0.004 | 418-425 | (R)AVTVERER(Q) |
| 1015.475 | 1015.484 | -0.009 | 432-439 | (R)DAYYVTQR(V) |
| 1299.638 | 1299.644 | -0.006 | 430-439 | (K)QRDAYYVTQR(V) |
| 1625.834 | 1625.839 | -0.006 | 432-444 | (R)DAYYVTQRVNLQR(Y) |
| 1681.911 | 1681.920 | -0.009 | 382-396 | (R)IYGLPVVVSEYFPAK(A) |
| 2213.135 | 2213.142 | -0.007 | 397-415 | (K)AADKEFAVIVYKDNFVMPR(Q) |
| 2309.186 | 2309.191 | -0.005 | 253-272 | (K)SFITDETEEDAIFSLLPLLR(K) |
| 2400.187 | 2400.186 | 0.001 | 275-297 | (R)LIEAHAVSIEEAFMTGNGTGQPK(G) |
| 2601.415 | 2601.432 | -0.017 | 182-204 | (R)IIRDLQKELVVGALFDELPMSSK(I) |
| 2692.404 | 2692.444 | -0.040 | 249-272 | (K)LAAKSFITDETEEDAIFSLLPLLR(K) |
| 3540.719 | 3540.709 | 0.010 | 344-374 | (K)LVLIVSMDAYYDLLEDEEWQDVAQVGNDAVK(L) |
| 3875.992 | 3876.044 | -0.052 | 382-415 | (R)IYGLPVVVSEYFPAKAADKEFAVIVYKDNFVMPR(Q) |
| 4413.183 | 4413.242 | -0.059 | 205-245 | (K)ILTMLVEPEAGKATWVDASKFGTDETVGDEVKGTLTEISFK(T) |
| **Band B** |  | | | |
| **1** M**SLQLLR**NTR **IFVSTVKTGH NK**TNTQEILV QDDISWGQDS NSTDITVNEA  **51** GPRPTRGSKR FNDSLNAAEW SFSTYILPYI NKEDQK**QIVP DYMLWHALSS**  **101** **GK**AINLEGNT GAHNNEANFM VNFKDNSYHE LAMLHIYILT DKAWSYIDSC  **151** QINQAEVNVD IEDIGRVTWS GNGNQLIPLD AQPFDPDELG IDDETYMTIQ  **201** SSYIKNKLTI LK**IKDMDSGK AYDIPITGGT FTINNNITYL TPNIMSRVNI**  **251 PIGSFTGAFE LTGSLTAYLN DK**SLGSMELY KDLIK**TLKVV NRFEIALVLG**  **301 GEYDEERPAA ILVAKQAHVN IPTIETDDVL GTSVEFKAIP SDLDAGDEGY**  **351 LGFSSKYTKT TINNLIVNGD GATNAVTAIT VK**SAGDTTTV NNSATLQMSV  **401** EVTPSSAKNK EVTWAITAGD AATIDPESGL LTADTTKTGE VTVEATAKDG  **451** SGVKGTKQIT VTAGG | | | | |
| 729.455 | 729.461 | -0.006 | 2-7 | (M)SLQLLR(N) |
| 1330.746 | 1330.747 | -0.001 | 11-22 | (R)IFVSTVKTGHNK(T) |
| 1892.916 | 1892.921 | -0.005 | 87-102 | (K)QIVPDYFLFAHALSSGK(A) (3 oxidations at M and W)) |
| 1941.896 | 1941.907 | -0.011 | 338-356 | (K)AIPSDLDAGDEGYLGFSSK(Y) |
| 2334.092 | 2334.113 | -0.021 | 338-359 | (K)AIPSDLDAGDEGYLGFSSKYTK(T) |
| 2413.213 | 2413.224 | -0.011 | 316-337 | (K)QAHVNIPTIETDDVLGTSVEFK(A) |
| 2628.348 | 2628.355 | -0.007 | 248-272 | (R)VNIPIGSFTGAFELTGSLTAYLNDK(S) |
| 2971.615 | 2971.625 | -0.010 | 289-315 | (K)VVNRFEIALVLGGEYDEERPAAILVAK(Q) |
| 3016.497 | 3016.508 | -0.011 | 221-247 | (K)AYDIPITGGTFTINNNITYLTPNIMSR(V) |
| 3313.841 | 3313.851 | -0.010 | 286-315 | (K)TLKVVNRFEIALVLGGEYDEERPAAILVAK(Q) |
| 3906.907 | 3906.925 | -0.018 | 213-247 | (K)IKDMDSGKAYDIPITGGTFTINNNITYLTPNIMSR(V) |
| 4615.302 | 4615.341 | -0.039 | 338-382 | (K)AIPSDLDAGDEGYLGFSSKYTKTTINNLIVNGDGATNAVTAITVK(S) |
| 4728.321 | 4728.320 | 0.001 | 316-359 | (K)QAHVNIPTIETDDVLGTSVEFKAIPSDLDAGDEGYLGFSSKYTK(T) |
| **Band C** |  | | | |
| **1** MAITTK**IIVQ QILNIDDTKA TASKFPR**YTV TLGNSISSIT ANELVSSIEA  **51** AAK**SAAAAKD SEIAAKTSEL NAKESENDAA ISAGASEASA TQSATSATQS**  **101 AASATK**SAES AAAAKTSETN SKTSETNAKT SEDASAAAAK ISETNAK**ASE**  **151 TNAAQSAADS SGFRNEAEIF SGQAAASASA AK**ISETNSKT SETNAKTSET  **201** NAAGSATSAS QSVAAIQGLK SDVEQLKSDT QAIKNSAVTE TTALKADVEQ  **251** LKSDTQAIKN SAVSEITTLK ADVEQLKTDT QGIKDSAVSD TTALKNQAAA  **301** SATQAGNSAI EAGQQASNAA GSANSSKAEA DRAK**AEADRA EVAANRGPDL**  **351 QPFPDVWIPF NDSLDMLAGY SPGYKKITVG EDVITMPSDK VVSFSRASNA**  **401 TYINKHGEFC IANIDEPRFE KQGLLIEGQR TNYITYSNDP ASLNTDKYRS**  **451 DVTYNIDKYG FAYATATVNE K**AQGEYPSLF YCETVNAINC QQNEYVSLSI  **501** RVKANLGIYI TPQFYLVGED GGLILGAR**SF ISCETGEVSS VVEGR**GTITH  **551** R**IYREDNGWL KVEATCKFVE RGGNSIGSVN YCRENDQPVQ VGDTISFCTP**  **601 QFEKGFCASS FIITGSTPAT RAVDYITIPA R**NNFSGTNIS LLAEVSINWD  **651** SFQLNNTYPM IIDNKPYYVE GK**AFVAEFDS TSATPYAYVV NEGGSTILSR**  **701 GYAFEKQFSP HVFGFIFSGN GDVTSFVNGN KGGTSHGSTW KGTDSDSLVE**  **751 IGGRPSDSTK LYGHIRNLRI WNRVLTDSQM R**EKV | | | | |
| 714.345 | 714.345 | 0.000 | 701-706 | (R)GYAFEK(Q) |
| 758.429 | 758.431 | -0.002 | 761-766 | (K)LYGHIR(N) |
| 877.482 | 877.489 | -0.007 | 20-27 | (K)ATASKFPR(Y) |
| 949.472 | 949.477 | -0.005 | 774-781 | (R)VLTDSQMR(E) |
| 971.542 | 971.553 | -0.009 | 767-773 | (R)NLRIWNR(V) |
| 1013.575 | 1013.573 | 0.002 | 422-430 | (K)QGLLIEGQR(T) |
| 1017.478 | 1017.475 | 0.003 | 732-741 | (K)GGTSHGSTWK(G) |
| 1118.620 | 1118.620 | 0.000 | 622-631 | (R)AVDYITIPAR(N) |
| 1232.642 | 1232.648 | -0.006 | 54-66 | (K)SAAAAKDSEIAAK(T) |
| 1283.572 | 1283.580 | -0.008 | 572-583 | (R)GGNSIGSVNYCR(E) |
| 1417.774 | 1417.779 | -0.005 | 419-430 | (R)FEKQGLLIEGQR(T) |
| 1518.783 | 1518.784 | -0.001 | 770-781 | (R)IWNRVLTDSQMR(E) |
| 1772.860 | 1772.863 | -0.003 | 605-621 | (K)GFCASSFIITGSTPATR(A) |
| 1814.856 | 1814.860 | -0.004 | 568-583 | (K)FVERGGNSIGSVNYCR(E) |
| 1842.848 | 1842.853 | -0.005 | 529-545 | (R)SFISCETGEVSSVVEGR(G) |
| 1920.910 | 1920.914 | -0.004 | 742-760 | (K)GTDSDSLVEIGGRPSDSTK(L) |
| 1981.977 | 1981.980 | -0.003 | 552-567 | (R)IYREDNGWLKVEATCK(F) |
| 2180.118 | 2180.126 | -0.008 | 377-396 | (K)ITVGEDVITMPSDKVVSFSR(A) |
| 2236.043 | 2236.052 | -0.009 | 431-449 | (R)TNYITYSNDPASLNTDKYR(S) |
| 2308.214 | 2308.221 | -0.007 | 376-396 | (K)KITVGEDVITMPSDKVVSFSR(A) |
| 2371.329 | 2371.334 | -0.005 | 7-27 | (K)IIVQQILNIDDTKATASKFPR(Y) |
| 2470.163 | 2470.177 | -0.014 | 450-471 | (R)SDVTYNIDKYGFAYATATVNEK(A) |
| 2660.321 | 2660.327 | -0.006 | 742-766 | (K)GTDSDSLVEIGGRPSDSTKLYGHIR(N) |
| 2703.273 | 2703.299 | -0.016 | 707-731 | (K)QFSPHVFGFIFSGNGDVTSFVNGNK(G) |
| 2952.412 | 2952.426 | -0.013 | 673-700 | (K)AFVAEFDSTSATPYAYVVNEGGSTILSR(G) |
| 3043.553 | 3043.555 | -0.002 | 742-769 | (K)GTDSDSLVEIGGRPSDSTKLYGHIRNLR(I) |
| 3373.553 | 3373.552 | 0.001 | 148-182 | (K)ASETNAAQSAADSSGFRNEAEIFSGQAAASASAAK(I) |
| 3397.611 | 3397.627 | -0.016 | 701-731 | (R)GYAFEKQFSPHVFGFIFSGNGDVTSFVNGNK(G) |
| 3814.753 | 3814.769 | -0.016 | 67-106 | (K)TSELNAKESENDAAISAGASEASATQSATSATQSAASATK(S) |
| 3918.949 | 3918.955 | -0.006 | 397-430 | (R)ASNATYINKHGEFCIANIDEPRFEKQGLLIEGQR(T) |
| 4192.932 | 4192.959 | -0.027 | 584-621 | (R)ENDQPVQVGDTISFCTPQFEKGFCASSFIITGSTPATR(A) |
| 4493.116 | 4493.150 | -0.034 | 335-375 | (K)AEADRAEVAANRGPDLQPFPDVWIPFNDSLDMLAGYSPGYK(K) |
| 4687.140 | 4687.211 | -0.071 | 431-471 | (R)TNYITYSNDPASLNTDKYRSDVTYNIDKYGFAYATATVNEK(A) |
| **Band D** |  | | | |
| **1** MTDKLIRELL IDVKQKGATR TAK**SIENVSD ALENAAAASE LTNEQLGKMP**  **51 R**TLYSIERAA DR**AAKSLTKM QASRGMAGIT K**SIDGIGDKL DYLAIQLIEV  **101** TDKLEIGFDG VSRSVKTMGN DVAAATEKVQ DRLYDTNRAL GGTAR**GFNDT**  **151 AGAAGR**ASRA IGNTSGSARG ATRDFAAMAK IGGSLPIMYA ALASNIFVLQ  **201** SAFEQLKLGD QLNRLEK**FGV IVGTQTGTPV QTLARSLQEA AGYAISFEEA**  **251 MRQASSASAY GFDAEQLNKF GLVARR**AAAV LGVDMTDALN RVIKGVSKQE  **301** IELLDELGVT IR**LNDAYADY VKQLNAANTG ITYNVNSLTT FQKQQAYANA**  **351 VIAESTKRFG YLDEVLR**ATP WEQFAANADA ALRKIQQAAA K**YLGPVIDAI**  **401 NTVFYTSQAS ISAEAAR**AQE KTNR**QIDPTN VGAVALSLAA SEEGYNKALD**  **451 MYKESLDKR**N KLKSEFDKRM EQADFYTKLA IRQVGEGIPV GLAAAGASEA  **501** NK**QFVAETAA MGLQVTR**LGK EVEDSTENLN AWKSAYQAAG AAAAK**ASPEF**  **551 QKQINLQR**DT TDPGAVYDFN STVLKGLTEQ QKAYNQTKKT ASDLANDIQN  **601** VAQNTDTAAK TSATLADAIK NIESLSLGTG KSADEYVKNL NLGYNTLSEM  **651** KTASQALSEY VK**LTGNETKN QLAVQQKIAD VYNQTKDKEK AQEAGR**R**LEL**  **701 QQLEEQEAAL RR**VLQTNQGN KAVEKEIEKI QLEKLKLTNQ GMEAQKKVKD  **751** YTDKILGVDR EIALLNNRTM TDTQYRLAQL NLELTIEKEK YEWYTKQADK  **801** QKEAEQSRRA QAQIEREIWK FRQDQIASMA AGREEEQQR**Q FTAKPLMGNA**  **851 ER**LQEQLKLY EDLKQKTLGN AAAQAEYNKK IAETR**AQLAG LR**AQR**NAEMQ**  **901 ASVGSSLGAV YTPTTGLSGE DKDFADMGNR** MASYDQAISK LSELNSEATA  **951** VAQSMGNLTN AMIQFSMGSL DTTSTIAAGM QTVASMIQYS TSQQVSAIDQ  **1001** AIAAEQKRDG KSEASKAKLK KLEAEKLKIQ QDAAKKQIII QTAVAVMQAA  **1051** TAVPYPFSIP LMVAAGLAGA LALAQASSAS SMSSIADSGA DTTSYLTLGE  **1101** RQK**NIDVSMS ANAGELSYIR GDQGIGSANS FVPR**AEGGNM YPGVSYQMGE  **1151** HGTEVVTPMV PMKATPNDEL KTSSNSTSGR PIILNISAMD AASFREFASS  **1201** NSGALRDAVE LALNENGASL KTLGNS | | | | |
| 728.431 | 728.441 | -0.010 | 886-892 | (R)AQLAGLR(A) |
| 771.441 | 771.447 | -0.006 | 553-558 | (K)QINLQR(D) |
| 1036.477 | 1036.480 | -0.003 | 146-156 | (R)GFNDTAGAAGR(A) |
| 1111.578 | 1111.578 | 0.000 | 359-367 | (R)FGYLDEVLR(A) |
| 1267.676 | 1267.679 | -0.003 | 358-367 | (K)RFGYLDEVLR(A) |
| 1404.683 | 1404.686 | -0.003 | 1121-1134 | (R)GDQGIGSANSFVPR(A) |
| 1478.734 | 1478.742 | -0.008 | 840-852 | (R)QFTAKPLMGNAER(L) |
| 1558.828 | 1558.833 | -0.005 | 546-558 | (K)ASPEFQKQINLQR(D) |
| 1637.835 | 1637.831 | 0.004 | 503-517 | (K)QFVAETAAMGLQVTR(L) |
| 1649.869 | 1649.860 | 0.009 | 344-358 | (K)QQAYANAVIAESTKR(F) |
| 1825.966 | 1825.976 | -0.010 | 697-711 | (R)RLELQQLEEQEAALR(R) |
| 1845.004 | 1845.022 | -0.018 | 218-235 | (K)FGVIVGTQTGTPVQTLAR(S) |
| 1888.869 | 1888.874 | -0.005 | 236-252 | (R)SLQEAAGYAISFEEAMR(Q) |
| 1982.042 | 1982.052 | -0.010 | 63-81 | (R)AAKSLTKMQASRGMAGITK(S) |
| 2164.081 | 2164.099 | -0.018 | 678-696 | (K)IADVYNQTKDKEKAQEAGR(R) |
| 2586.293 | 2586.305 | -0.012 | 253-276 | (R)QASSASAYGFDAEQLNKFGLVARR(A) |
| 2757.393 | 2757.409 | -0.016 | 392-417 | (K)YLGPVIDAINTVFYTSQASISAEAAR(A) |
| 2952.428 | 2952.500 | -0.072 | 359-384 | (R)FGYLDEVLRATPWEQFAANADAALRK(I) |
| 2974.394 | 2974.442 | -0.048 | 24-51 | (K)SIENVSDALENAAAASELTNEQLGKMPR(T) |
| 3241.570 | 3241.554 | -0.016 | 1104-1134 | (K)NIDVSMSANAGELSYIRGDQGIGSANSFVPR(A) |
| 3451.701 | 3451.716 | -0.015 | 503-533 | (K)QFVAETAAMGLQVTRLGKEVEDSTENLNAWK(S) |
| 3608.623 | 3608.611 | 0.012 | 896-930 | (R)NAEMQASVGSSLGAVYTPTTGLSGEDKDFADMGNR(M) |
| 3812.898 | 3812.901 | -0.003 | 425-459 | (R)QIDPTNVGAVALSLAASEEGYNKALDMYKESLDKR(N) |

^a^ Phage AKFV33 was purified by two round CsCl-density gradient centrifugation and separated in 10% SDS-PAGE gel.

^b^ Sequence in red ink indicates amino acids identified by the MALDI QqTOF MS and MS/MS analysis
